# Differentiated characteristics, sustainability performance and preferences among small-scale aquaculture producers: implications for sustainable intensification

**DOI:** 10.1007/s11625-025-01703-w

**Published:** 2025-08-01

**Authors:** Eric Brako Dompreh, Quanli Wang, Jie Su, Rodolfo Dam Lam, Benoy Kumar Barman, Cristiano M. Rossignoli, Alexandros Gasparatos

**Affiliations:** 1 WorldFish, Jalan Batu Maung, Batu Maung, 11960 Bayan Lepas, Penang Malaysia; 2 Institute for Future Initiatives (IFI), The University of Tokyo, 7-3-1 Hongo, Bunkyo-ku, Tokyo, 113-8654 Japan; 3 WorldFish, Bangladesh Office, House 22B, Road 7, Block-F, Banani, Dhaka 1213 Bangladesh; 4 Institute for the Advanced Study of Sustainability (UNU-IAS), United Nations University, 5-53-70 Jingumae, Shibuya-ku, Tokyo, 150-8925 Japan

**Keywords:** Aquaculture, Sustainable intensification, Improved fish strains, Food security, Food system characterization, Choice experiment, Bangladesh

## Abstract

Inland aquaculture is becoming an important source of animal-based protein in many low- and middle-income countries of Asia. In several of these countries such as Bangladesh the inland aquaculture sector is dominated by small-scale producers that rely on unimproved production practices. However, the heterogeneity of these producers is not well understood, leading to assumptions of low variability in both the underlying characteristics of aquaculture production systems, as well as their sustainability performance. This in turn can  lead to the ineffective design and implementation of interventions and policies for the sustainable intensification of the sector. This study explores the differentiated characteristics, sustainability performance and preferences among carp producers in Bangladesh. We focus on Bangladesh as it is the 5th largest inland aquaculture producer globally, with the sector contributing significantly to livelihoods and food security. We undertake comprehensive in person surveys with 4540 carp producers across 54 regions of the country. We use 18 production variables to develop a nuanced typology of carp production systems using Principal Component Analysis (PCA) and Hierarchical Clustering Approach (HCA). We then assess whether these production systems have significantly different sustainability performance across eight socioeconomic and environmental indicators. Finally, we elicit the preferences of producers for improved carp species through three choice experiments. Overall, we identify four major types of  carp production systems, characterized by very different production characteristics, sustainability performance and preferences for improved fish species attributes. Collectively, our results provide a nuanced picture of the carp aquaculture sector in Bangladesh, which moves beyond simple binaries (e.g., commercial vs. subsistence; intensive vs. extensive; large-scale vs. small-scale). This information can inform the development and implementation of fit-for-purpose interventions for the sustainable intensification of the sector.

**Supplementary Information:**

The online version contains supplementary material available at 10.1007/s11625-025-01703-w.

## Introduction

The demand and consumption of animal-based protein has rapidly increased globally in the past decades (Golden et al. 2021; Henriksson et al. 2018) largely fueled by population growth and dietary changes (Alexander et al. 2016; Dam Lam et al. 2022; Weindl et al. 2017), among other factors. This increased demand has been met through the rapid expansion of industrialized terrestrial livestock production systems that are largely unsustainable in terms of greenhouse gases (GHGs) emissions (Henriksson et al. 2021, 2018), pollution (Edwards 2015), and land use change and biodiversity loss (Diana 2009). Although it is crucial to boost sustainably the supply of animal-based protein in some parts of the world both to meet increasing demand and enhance household nutrition and food security (Beal et al. 2023; Béné et al. 2016), the expansion of terrestrial livestock production systems is not always possible or sustainable due to production constraints (e.g., land/water scarcity), cultural reasons (e.g., religious sensibilities), and/or environmental concerns (Troell et al. 2014; Willett et al. 2019).

In this context, it has been argued that aquatic food systems can cater for a substantial portion of increased animal-based protein demand through relatively more sustainable production models (Crona et al. 2023; Gephart et al. 2021). Among the diverse aquatic food systems, small-scale inland aquaculture shows particular promise (Zhang et al. 2022). Despite some negative environment impacts such as high nutrient loading (Verdegem 2013) and GHGs emissions (Chen et al. 2023), small-scale inland aquaculture has been associated with several positive socioeconomic outcomes. These include nutritional and food security (Dam Lam et al. 2022; Wang et al. 2024), and rural livelihoods and poverty alleviation (Béné et al. 2016; Toufique and Belton 2014), especially in some low- and middle-income countries (FAO 2024). Statistics suggest that inland aquaculture likely had the highest contribution to the global production increases and food security improvements from aquatic food systems in the past decades (particularly in Asia), mainly through polyculture systems of diverse carps and other fish species (e.g., tilapia, catfish) at the household and small/medium commercial enterprise level for local and regional consumption (Naylor et al. 2021). However, in many areas with high potential for small-scale inland aquaculture, the actual production systems are still characterized by  high reliance on rudimentary production techniques (Wang et al. 2024) and low productivity (Dey et al. 2010). This in turn curtails the sustainable development outcomes of these aquatic food systems (Belton 2014; Kawarazuka and Béné 2010).

Arguably it is crucial to transform inland aquaculture systems if we are to meet the current and future demand for fish and other aquatic products. The sustainable intensification of small-scale aquaculture systems in low- and middle-income countries where they underperform holds tremendous potential (Hamilton et al. 2022a)  Many national and international organizations have already committied substantial financial investments in achieving sustainable intensification through interventions promoting improved inland aquaculture production technologies and management practices (Hamilton et al. 2022b) such as better management practices (BMPs) (Dompreh et al. 2023; Henriksson et al. 2021) and improved fish strains (Nguyen 2016). However, their adoption, sustained use and ultimate success is not automatic, and sometimes fails to yield the expected outcomes. A key reason is the assumption that small-scale inland aquaculture producers have homogenous needs, preferences and capacities, which cannot be further from reality considering that they are one of the most diverse and dynamic segments of aquatic food systems globally (Short et al. 2021). This assumption is often caused and perpetuated by the lack of high-quality benchmarking data about the actual characteristics, performance and needs of small-scale aquaculture producers, likely contributing to poor intervention design and implementation. It is not uncommon for such interventions to adopt blanket beneficiary targeting approaches, essentially promoting solutions that do not reflect the actual needs or capabilities of all targeted producers, and eventually underperforming at best in terms of productivity and sustainability or being abandoned at worst (Belton and Little 2011; Henriksson et al. 2021). This lack of fine-grained benchmarking data about small-scale producers can be particularly problematic in low- and middle-income countries where the aquaculture sector is very dynamic and/or experiences fast change (Short et al. 2021).

Bangladesh experienced many of the circumstances outlined above. On the demand side, aquatic food is essential for meeting the growing demand for sustainable animal-based protein. Annual per capita fish consumption is higher than the global average, and accounts for as much as 60% of national animal-based protein consumption (FAO 2024). On the production side, aquaculture output is very high, with Bangladesh ranking as the 5th largest aquaculture producer globally (FAO 2024). The sector has expanded substantially in the past decade and plays a major role for the livelihoods and food security of a large fraction of the population (DoF, 2022). Inland ponds are now the single largest source of aquatic food (Figure S1, Supplementary Material), and account for 94% of the total national aquaculture output (Hernandez et al. 2018). Roughly 4.3 million households (20% of the rural population) operate at least one homestead pond that is usually small in size (0.08–0.10 ha on average) and contain mainly carp, pangasius catfish, and tilapia species (Dam Lam et al. 2022; Rossignoli et al. 2023). As a result the inland aquaculture sector has been central in many national policies and strategic plans (WorldFish 2024). It was identified as important for national economic growth in the 8th Five-Year Plan (8FYP) (GED 2020a) and in efforts to transition to an upper middle–income country by 2031 and a high-income country by 2041 (GED 2020b). Furthermore, inland aquaculture was mainstreamed in the recent National Adaptation Plan (MEFCC 2022).

Carp is by far the most dominant group of aquaculture species in the country, accounting for 55.4% of the total output (Figure S2-3, Supplementary Material) (DoF 2023). Carp production is not geographically concentrated, but occurs throughout most of the country (Figure S4, Supplementary Material) and is overwhelmingly geared for household self-consumption and/or the national market (Rossignoli et al. 2023). Carps are especially valued among small-scale producers due to their fast growth, high market value and resonance with cultural preferences, which position them as their preferred option (Belton and Azad 2012; Karim et al. 2016; Rossignoli et al. 2023).

However, many small-scale producers in Bangladesh are still plagued by reliance on unimproved technologies and lack of access to high-quality fish seed, which take a toll on the performance and sustainability of the sector (Karim et al. 2016; Rossignoli et al. 2023). Public and  (increasingly) private organizations have been promoting improved aquaculture practices (Naylor et al. 2023), including BMPs and improved carp strains (e.g., G3 rohu) that could potentially have transformative effects for the sector (Hamilton et al. 2022b). However, such investment decisions and promotion efforts are undermined by the lack of comprehensive and high-quality data and benchmarks about the differentiated characteristics of inland aquaculture systems in the country (Dompreh et al. 2023; Henriksson et al. 2018; Rahman et al. 2022). This is especially the case for carp aquaculture systems that are particularly prevalent across the country but also very diverse and dynamic.

The aim of this study is to disentangle differentiation in the characteristics, sustainability performance and preferences of carp producers in Bangladesh. Using a comprehensive survey of 4,540 producers in 54 regions across the country we develop a nuanced typology of carp production systems through Principal Component Analysis (PCA) and Hierarchical Clustering Approach (HCA). We then assess whether these production systems have significantly different sustainability performance across eight environmental and socioeconomic indicators. Finally, we elicit the preferences of producers for improved carp species through three choice experiments considering that genetic improvement is a promising avenue for sustainable aquaculture intensification (FAO 2024; Houston et al. 2020).

## Methodology

### Research approach

The study presented in this paper consisted of six main steps: (a) study site identification (Step 1), (b) survey and sampling design (Step 2), (c) data collection and cleaning (Step 3), and (d) data analysis (Steps 4–6). Data analysis aimed to (a) develop a typology of carp production systems (Step 4) (b) compare the socioeconomic and environmental performance of  carp production systems (Step 5), and (c)  elicit and compare producers' preferences for improved carp species (Step 6).

#### Step 1: site identification

Government statistics about aquaculture production in Bangladesh tend to be highly aggregated. The available data is generally available only for fish output and only at the highest levels of administration such as the Division (*n* = 8) or the District (*n* = 64). However, these administrative levels are generally large in terms of land and population size, and can contain numerous zones where inland aquaculture might be already performed or be viable in future. The upazila level (roughly a county) (*n* = 495) is a more appropriate level of analysis, but the available aquaculture data is not so finely grained in terms of aquaculture output or farm production characteristics.

In other words from the available government statistics it is not always clear in which upazilas carp aquaculture occurs, or what are the characteristics and performance of carp production systems. Due to this lack of reliable, comprehensive and spatially explicit information, we identified study upazilas through a combination of expert consultations, suitability analysis, and government statistics (where available).

First, we conducted 8 regional expert workshops with 215 experts from around Bangladesh. Experts were selected on the basis of their knowledge about carp aquaculture in their respective region, and represented diverse organizations from government, academia, civil society, international organizations and the private sector (for the composition of each workshop refer to: Rossignoli et al. (2023)). These workshops enabled us to synthesise expert perspectives about: (a) which upazilas currently have high carp production (hotspots), and (b) which upazilas have such potential. Furthermore, we elicited information about which characteristics of carp species are valued among producers, and what are the challenges and opportunities facing the carp aquaculture sector. Detailed information about the design, composition, implementation and outcomes of these workshops can be found elsewhere (Rossignoli et al. 2023). Further information was gathered through expert consulation with regional fisheries offices and relevant national aquaculture and fisheries statistics (DoF, 2022).

Through this process we selected 54 upazilas, of which 30 are currently considered carp aquaculture hotspots, and 24 upazilas have this potential (Figure S9 and Table S3, Supplementary Material). These upazilas span the entire country apart from the eastern part of Chittagong Division, which is generally characterized by low inland aquaculture output (DoF, 2022) and suitability (see next paragraph).

e verified upazila selection through an aquaculture suitability analysis that used spatially explicit secondary data to develop a composite suitability index accross ten dimensions of water quality, soil quality and infrastructure, as identified through a literature review. Box S1, Tables S1-2 and Figures S11-15 in the Supplementary Material outline the methodological approach, underlying datasets and intermediate results of the aquaculture suitability analysis. The key output was an aquaculture suitability map (30 × 30 m resolution) that identified areas with high, moderate and low potential for aquaculture (Figure S14, Supplementary Material). We then overlayed the upazilas identified from the expert consultation process outlined above to ensure that selected areas had characteristics that made them appropriate for aquaculture. This analysis showed that all selected upazilas had overwhelmingly high and moderate aquaculture suitability.

#### Step 2: survey and sampling protocol

The main data collection instrument was a structured household survey, consisting of two major components. The first component elicited information about the characteristics and performance of carp aquaculture systems. The second component elicited information about the preferences of carp producers about improved carp strains.

The central element of the characterization and performance component was a comprehensive module about fish production, commercialization and knowledge. In particular, for each pond operated by the responding households (i.e., carp producers) we captured for the previous production cycle the fish species produced, sold (raw or processed), gifted and consumed, as well as the generated income. We also captured comprehensive information about all aquaculture inputs and expenditures (e.g., feed, seed, labor, materials). Other survey modules extracted information about the demographic composition, socioeconomic status and food security of the respondent households. The survey contained overwhelmingly closed ended question, for which respondents had to either provide actual numerical values or enumerators had to assign preexisting codes based on the answer of the respondent. A comprehensive description of the survey questionnaire can be found elsewhere (Gasparatos et al. 2024).

The preferences component contained three discrete choice experiments (DCEs) with each respondent household. DCEs have gained popularity for eliciting how people value multiple attributes when making decisions through hypothetical scenarios that involve tradeoffs between choices (Aizaki 2012; Kjær 2005; Oleson et al. 2015). We opted using a series of DCEs rather than another perception elicitation mechanism (e.g., ranking exercise) given the ability of DCEs to elicit more accurately the choices respondents must make in real life (Manyise et al. 2024). The three DCEs elicited preferences across the following themes: (a) aquaculture productivity (DCE1), (b) fish traits (DCE2), and (c) pond support systems and management (DCE3). Through consultation with experts involved in carp improvement (Hamilton et al. 2021, 2022b) (incl. in Bangladesh) we developed for each DCE scenarios (*n* = 2), attributes each (*n* = 5), and attribute levels in line with the aforementioned themes. For DCE1, the attributes cover productivity aspects such as feed conversion rate (FCR), fish survival rate, input cost, fish growth rate, and fingerling cost. For DCE 2, attributes cover fish traits and preferences such as bone density, taste/fat content, fish appearance, fish size, and fingerling cost (Ethin et al. 2019; Islam et al. 2011; Ljubojević et al. 2017). We note that as taste is a highly subjective attribute, we use fat content as a support descriptor to create some consistency between respondents. This reflects the fact that other widely produced and consumed inland aquaculture species in Bangladesh such as tilapia are generally not considered as good tasting as traditional carps due to their lower fat content. For DCE3, the attributes cover aquaculture management aspects such as hired labor, government extension support, ecosystem-based management, network and collaborations, and fingerling cost (Alam et al. 2022; Kucukgul 2008; Selim et al. 2021). We use an orthogonal design to reduce the number of experiments, while providing the ability to estimate the main effects estimated with precision (Grömping 2018; Holmes et al. 2017; Walker et al. 2018). We used the *Support.CEs* package in R (version 3.6.3) for the orthogonal design for each DCE and choice cards generation (Aizaki 2012). For each DCE, we presented a total of 16 questions to each respondent. Each question requested respondents to choose their preferred option between three possible alternatives, consisting of two scenarios with the attributes mentioned above based on the orthogonal design, and an opt-out option of “none of the options presented”. Refer to Figure S16-S18 (Supplementary Material) for examples of each DCE.

Due to the lack of a centralized, comprehensive and spatially explicit database of carp producers in Bangladesh (or specific regions), we followed a multistage approach to identify respondents and randomize their selection. Initially, we contacted and visited all Upazila Fisheries Office to inquire: (a) whether comprehensive carp producer lists were available for each study upazila, (b) what were the main areas of carp production in each study upazila (e.g., para, unions). In most upazilas such lists were not available, while in the few cases that such lists existed they were quite outdated (8–10 years old). For this reason, we proceeded to generate new carp producer lists. First, we randomly selected para and/or unions in each upazila from the ones indicated by the respective Upazila Fisheries Offices. Second, we visited these para/unions and registered every available carp farmer we could find, and then moved to another randomly selected area until we had a list of 130–150 carp producers for each upazila. This resulted in a database of 7540 carp producers across all upazilas. Third, we randomly selected farmers from each upazila list for data collection.

There is no reliable estimate about the number of households engaged inland aquaculture in Bangladesh, let alone carp aquaculture. However, national statistics estimate approximately 2.5 million inland aquaculture ponds (DoF 2023), implying that a maximum of 2.5 million households are engaged in inland aquaculture in Bangladesh (some households own multiple ponds, but this is the exception rather than rule). Using a confidence level of 97% and following the estimations by Taherdoost (2017), we estimated that a minimum sample size of 1067 households (about 20 in each of the 54 selected upazilas). We increased substantially the number of respondents to enhance statistical generalization by aiming at interviewing 84 carp producers in each upazila, for a final sample was 4540 producers. Table S3 (Supplementary Material) contains the producer numbers for each upazila.

The Institutional Review Board of the Institute of Health Economics (IHE-IRB), which is approved by the U.S. Department of Health and Human Services Federalwide Assurance (FWA), No. FWA00026031, provided ethical approval of the protocol.

#### Step 3: data collection and cleaning

The survey was translated in Bengali and digitized in tablets for in person data collection using 44 well trained enumerators. The local consultancy Development Research Initiative (dRI) supervised the data collection on the ground**.** The sheer size of the survey needed to achieve the proper characterization (4540 households in 54 upazilas across the country), required a large team that was beyond the manpower and logistical ability of the author team to supervise effectively.

After the initial training of the enumerators and supervisors (supervised by members of the research team), the enumerators conducted pilot surveys with aquaculture producers in selected study areas. This piloting further helped to identify possible problems with the framing of the questions (e.g., clarity, sensitivity) and the ranges used for the coding of the categorical questions. Through iterative interactive discussions between the supervisors, enumerators and the research team the survey protocol was revised slightly before the final deployment.

The full household survey was conducted between 16th November 2021 and 28th February 2022. The survey targeted the person responsible for pond operation in each household, as we needed to capture very technical information about pond operation, and accurate quantitative estimates about fish output, fish commercialisation, and household budgets. In most cases this was the household head, which was in most cases the male spouse (Figure S9a-b, Supplementary Material).

To ensure the quality of the collected data we implemented various quality assurance mechanisms to reduce non-sampling errors, namely (a) select an experienced data collection team; (b) design and digitise carefully the questionnaire to capture accurately characteristics/performance, and assist respondent recollection for sensitive variables (see also “Challenges and limitations”); (c) train enumerators to adhere properly to the study protocol; (d) revise iteratively the questionnaire prior to implementation using insights from training and piloting; (e) check daily all collected surveys for completeness and quality, and provide timely and constant feedback to enumerators. More information about the data collection protocol and the quality assurance mechanisms can be found elsewhere (Gasparatos et al. 2024).

Following the data collection, the research team conducted a thorough data cleaning process to identify, correct and remove errors in the datasets. First, we went through the entire digitized database to ensure that we captured all variables correctly, removing surveys with any incomplete data. Second, an outlier analysis identified surveys with extreme values using the upper bound and lower bound method. Overall, we identified and removed 329 outlier surveys, resulting in a final sample of 4211 surveys for the full analysis.

#### Step 4: clustering analysis and typology development

For the characterization and group delineation analysis, we use a combination of the PCA (Greenacre et al. 2022; Lu et al. 2011) and the HCA (Martín-López et al. 2017; Panneerselvam et al. 2023). Such techniques have been used to identify differences between producers engaging in different farming activities in developing contexts (Alvarez et al. 2018; Martín-López et al. 2017).

First, we identified 28 different variables that are important for describing/characterizing inland aquaculture systems for the clustering. We identified these variables through a literature review and expert consultation, and secondarily from the insights gained from the expert workshops. The variables covered different farm characteristics that could affect aquaculture production, including location, pond characteristics, market orientation, fish species, fish yields, feeding regimes, fish seed selection, technology adoption, labor, aquaculture assets and investment, access to aquaculture information, and engagement in producers’ groups. Collectively, these variables covered comprehensively the four dimensions of inputs/assets, markets/demand, management/institutions, and specialization/diversification that have been suggested as important elements of differentiation in studies that have developed heuristics of small-scale actors in aquatic food systems (Aung et al. 2021; Belton and Azad 2012; Jahan et al. 2015; Wang et al. 2024). The full list of variables is included in Table S4-S5 (Supplementary Material).

Second, we performed a correlation analysis to establish the levels of relationship between the 28 variables, and whether all should be retained for the PCA. If two variables were highly correlated, then one was excluded from the subsequent PCA. As a rule of thumb, correlation coefficients of < 0.5 suggest little commonality between two variables. In total, after the correlation analysis 18 variables were eventually used for the PCA out of the 28 variables initially identified (Table S4, Supplementary Material). Table S6 (Supplementary Material) contains the correlation analysis and related coefficients for the 18 variables used for the clustering analysis.

Third, we conduct a PCA to reduce the dimensionality of the data considering that the analytical variables had a large diversity of different dimensions (Greenacre et al. 2022). The PCA yielded 18 components (Table S7-8, Supplementary Material). We retain for further analysis the six components with eigen values greater than or equal to 1, as they have a higher retention of sample characteristics and can best describe the set of data (Lu et al. 2011) (Table S7, Supplementary Material). We estimate the Kaiser–Meyer–Olkin (KMO) indicator, which is a measure of sampling adequacy that takes values between 0 and 1. A common rule of thumb is that values < 0.5 have little in common to warrant a PCA, but as our KMO estimation yielded 0.764, we infer that a PCA was warranted (Kaiser 1974; Regmi and Jones 2020) (Table S9, Supplementary Material).

Fourth, through a HCA we used the 6 components generated in the PCA to cluster the aquaculture farmers into different clusters/groups with similar production characteristics (Martín-López et al. 2017; Panneerselvam et al. 2023). Using the Calinski and Harabasz pseudo-*F* index and the Duda-Hart Je(2)/Je(1) index, we determined the optimum number of clusters that best describes the data (Table S10-S11, Supplementary Material). Higher values indicate more distinct clustering. Presented along with the Duda-Hart Je(2)/Je(1) index is the pseudo-T-squared, where lower values suggest more distinct clustering (Milligan and Cooper 1985). In this study, the value of the Calinski and Harabasz pseudo-*F* index estimation is 614.874 (Table S10, Supplementary Material), which suggests that the optimum number of clusters is four. Also, the value of the Duda-Hart Je(2)/Je(1) index is 0.8996, which corresponds to a lower pseudo-T-squared value of 235.23 (Table S11, Supplementary Material). This confirms that four distinct clusters is appropriate for this study (Table S10-S11, Supplementary Material).

Fifth, we conduct one-way ANOVA tests to estimate whether the differences for each variable used or omitted from the clustering analysis were statistically significant between groups. We conducted these pairwise comparisons to further verify whether the identified groups represented clearly different production systems. Figures S5-S6 in the Supplementary Material contain the outcomes of the one-way ANOVA tests for the variables included and excluded respectively from the clustering analysis.

Here we need to acknowledge that there are different clustering methods, which generally have the common aim of finding patterns within large datasets (Guevara-Viejó et al. 2021; Rasool and Abler 2023). Broadly speaking the choice of clustering method can affect clustering identification (Ezugwu et al. 2022), and should depend on the aims of the study and the characteristics of the data being analyzed.

For the purpose of this study, we chose PCA and HCA, as our primary aim was to identify the possible differentiation in the characteristics of carp aquaculture systems, without pre-determining the number of clusters. These techniques can reduce data dimensionality while preserving variation and to accommodate hierarchical relationships in household production patterns. This makes them well-suited for this type of study. Yet, HCA may be highly sensitive to outliers (Almeida et al. 2007), which is the reason we removed outliers from the analysis (see above).

Alternative clustering methods that were considered for this study (i.e., for segmenting carp aquaculture systems based on production characteristics) were the k-means clustering and latent class analysis (LCA). K-means clustering is widely used in agricultural studies for its efficiency in handling large datasets and clearly defined cluster separation (Shedthi et al. 2017). However, it assumes spherical clusters, which may not be ideal for complex, multidimensional data (Jolliffe and Cadima 2016; Kaur et al. 2021), such as the ones collected in this study and considered necessary for the proper characterization of aquaculture systems. Furthermore, k-means performs better for continuous numerical continuous variables (Ikotun et al. 2023), while the HCA used here performs better for the mix of continuous and categorical variables in our analysis. PCA, on the other hand, allows for the identification of latent subgroups based on probabilistic modeling, and has also been used in agricultural studies (Granato et al. 2018; Otitoju and Enete 2016). However, its categorical data assumption and reliance on model fit criteria are major limitations that prevented its application in our study (Vermunt and Magidson 2002).

#### Step 5: sustainability performance and experience of climatic shocks

We estimated the sustainability performance of the different groups across eight dimensions: (a) fish sales, (b) aquaculture Benefit–Cost Ratio (BCR) (c) household income, (d) multidimensional poverty, (e) dietary diversity, (f) fish self-consumption, (g) phosphorus use efficiency and (h) nitrogen use efficiency. Collectively, these dimensions provide a good cross section of the economic, social, and environmental sustainability impacts of food systems, and particularly inland aquaculture systems (Wang et al. 2023; Dompreh et al. 2023; Dam Lam et al. 2022). Table S12 (Supplementary Material) outlines how each of these indicators is estimated.

The three indicators of economic sustainability cover aquaculture profitability (fish sales per unit area), cost-effectiveness of aquaculture (BCR) and household income (income per person). For the first, we aggregate all income streams from fish sales for all harvested fish species from all operational household ponds in the past 12 months, and then divide the sum by the total pond area. For the second, we aggregate all incomes and costs related to aquaculture incurred over the past 12 months, and divide the income with the costs. A BCR of > 1 indicates that the economic benefits of aquaculture surpass its costs, signifying that the households' aquaculture activities are cost-effective. For the third, we aggregate all income streams (including from aquaculture, other farming/livestock activities, off-farm activities) in the past 12 months, and divide by the number of household members.

The three indicators of social sustainability cover aspects of nutrition (fish self-consumption by household members, household dietary diversity) and multi-dimensional poverty (deprivation scores). We estimated fish self-consumption by aggregating the amount of all consumed fish in the past 12 months produced in the households’ ponds, divided by number of household members within the household. This is for all fish species from all operational ponds operated by the household. We captured dietary diversity through the Food Consumption Score (FCS), which is a standardized composite proxy measure of nutritional quality at the household level (WFP 2008). The FCS captures the frequency of household consumption for eight food items over the seven days prior to the survey, weighted based on their relative nutritional value (Table S13, Supplementary Material). Deprivation scores at household-level capture through a standardized metric deprivation across the three dimensions of education (two sub-variables), health (two sub-variables) and living standards (six sub-variables), using different weights of each sub-variables (Table S14, Supplementary Material) (Alkire and Santos 2014).

The two indicators of environmental sustainability cover nutrient use efficiency, particularly of phosphorus and nitrogen (Wang et al. 2023). In inland aquaculture systems, feed and fertilizers are the primary sources/inputs of nitrogen and phosphorus to stimulate the growth of aquatic animals and vegetation. We quantified nitrogen and phosphorus use efficiency (%)by calculating the ratio of nitrogen and phosphorus output (in kg, sourced from the fish) in relation to the input (in kg, obtained from the total amount of feed and fertilizer use) (Wang et al. 2023). Data about aquaculture inputs and outputs came from survey questions pertaining to the annual input of different types of feed (e.g., commercial pellet feeds, rice bran, maize bran) and fertilizers (e.g., cow dung, poultry droppings, urea). Table S15 (Supplementary Material) contains the coefficients utilized for estimating nitrogen and phosphorus input and output, which include the nitrogen and phosphorus compositions of feeds, fertilizers, and various fish species. To reflect the context-specificity of the Bangladesh aquaculture sector, all these data are derived from Bangladesh-specific publications and reports (Barman and Karim 2007; Bogard et al. 2015; Shaheen et al. 2013; Tacon et al. 2009; Tacon and Metian 2013).

We estimated the experience and effects of climatic shocks through semi-quantitative questions following a two-stage process. First, we identified which types of climatic shocks were the most commonly experienced by respondents over the 5 years prior to the survey (Table S19, Supplementary Material). Subsequently, we selected floods and storms for further analysis, as they were the two most commonly experienced shocks in terms of the percentage of households experiencing them. For each household experiencing floods and storms in the past 5 years we estimated their perceived effects on aquaculture output and household income. Perceived effects were captured through a 4-level Likert Scale (1 = no effect, 2 = reduced slightly, 3 = reduced moderately, 4 = reduced significantly).

#### Step 6: choice experiment

The standard Random Utility Modeling framework (McFadden 1974) and the Lancaster theory of value (Lancaster 1966) form the analytical basis of choice experiments. Previous studies of discrete choices have used the Multinomial Logit Model (Hensher et al. 2005), which rests on the assumption that respondents have homogeneous preferences and that there is independence of irrelevant alternatives (IIA assumption). However, the IIA assumption is generally rejected for the unobserved preferences heterogeneity among respondents (Louviere et al. 2010). Thus, here we apply a random parameter logit model (RPL) to account for preference heterogeneity and help relax the IIA. Rather than calculating a single probability for each alternative, the RPL estimates the choice probability for each random draw taken from the assumed probability distributions. We estimate the RPL at 500 times Modified Latin Hypercube Sampling (MLHS) draws (Hess et al. 2006).

We assume a log-normal distribution for the coefficient of the cost attribute and a normal distribution for the coefficients of other attributes. This assumption is reasonable since it restricts cost coefficients to be negative for all respondents (Giergiczny et al. 2012) and has been widely used in choice experiments (Daly et al. 2012; Mariel et al. 2021a). Furthermore, an Alternative Specific Constant (ASC) is included in the model to capture preferences for the “no change” scenario (i.e., the status quo).

Considering the above, the utility obtained by respondent *n* from the alternative *j* could be expressed as shown in Eq. 1:

1 $${U}_{nj}={V}_{nj}+{\varepsilon }_{nj}={\alpha }_{j}{ASC}_{j}+\sum_{k}{\beta }_{nk}{X}_{njk}+\sum_{k}{\gamma }_{nk}{X}_{njk}{C}_{n}+{\mu }_{nj}{ASC}_{nj}{C}_{n}{\epsilon }_{nj},$$

where ${V}_{ij}$ denotes the deterministic component of utility, ${\epsilon }_{ij}$ denotes the unobserved component, ${X}_{njk}$ indicates the attributes, ${\beta }_{nk}$ is the coefficient revealing the aggregate preference of an individual ${\beta }_{k}$, ${stde}_{nk}$ denotes the standard deviation from the average preference in the RPL. For the random portion of the utility, interactions of characteristics of individual ${C}_{n}$ with attributes ${X}_{njk}$ or alternative specific constants ${ASC}_{nj}$, ${\gamma }_{nk}$ and ${\mu }_{nj}$ are interpreted as the explanation of the heterogeneity of preferences (Louviere et al. 2010).

We use estimated parameters from the model to derive the marginal willingness-to-pay (MWTP) for the fingerlings of improved strains. Following Mariel et al. (2021a, b) we use Eq. 2 to estimate the MWTP and employ the commonly applied delta model to compute the standard errors (Kruse and Atkinson 2022):

2 $${\mathrm{MWTP}}_{k}=-\frac{{\beta }_{k}}{\mathrm{exp}\left({\beta }_{\mathrm{cost}}+\frac{{{stde}_{\mathrm{cost}}}^{2}}{2}\right)}.$$

### Challenges and limitations

Despite the robustness of the findings and the large scope of the study in terms of geographic coverage and sample size (to the best of the authors’ knowledge this is one of the largest aquaculture studies of this type in low- and middle-income countries), there are certain challenges and limitations that should be noted when generalizing the findings.

First, due to the lack of a comprehensive and spatially explicit centralized database of carp production and individual producers in Bangladesh, the research team had to identify (a) study areas with substantial carp aquaculture and future potential, and (b) individual carp producers in the selected study areas. In order to reduce to the extent possible any uncertainties and biases we followed very structured study selection approach.

For (a) we followed a two-stage process relying on expert workshops and GIS-based aquaculture suitability analysis, triangulated to the extent possible with secondary data from relevant government statistics (see Step 2 above). Although relying on expert knowledge might have inserted certain biases and uncertainties when identifying appropriate study areas, we believe that the large number of involved experts (215 from the entire country) and the combination of these approaches resulted indeed in the identification of the most appropriate study areas.

For (b) we must stress that we did not identify all carp producers in a given upazila as this study has not aspired (or designed for that matter) to be a census of all carp producers in the country, as this would have been prohibitively resource consuming. The most important consideration for the scope of this study was to ensure the identification and selection of a well randomized sample of carp producers in each study upazila. As outlined in Step 2 above we followed a multi-stage process to identify and randomize the selected respondents. However, it is likely that in some upazilas randomization might not have been perfect and some selection biases might have occurred.

Considering this lack of spatially disaggregated national statistics about the main areas of carp production in Bangladesh, as well as the number, output, and characteristics of carp aquaculture producers, it is not possible to formally test the representativeness of the selected sample. However, we outlined above several processes to enable the robust study area selection, sampling framework development and respondent randomisation. These collectively increases confidence that the final sample was a good representation of the on-ground differentiation of carp producers, as also identified in the expert workshops (Rossignoli et al. 2023) and other studies in Bangladesh (Hossain et al. 2022; Jahan et al. 2015).

Second, some of the key variables used in this study such as fish production/sales and income had a 12-month recall period. Such long periods may cause recollection difficulties to some respondents, possibly underestimating the levels of such variables (Beegle et al. 2012; Deininger et al. 2012; Wang et al. 2024). We aimed to reduce errors due to recollection difficulties through (a) the careful design and digitization of the survey, (b) the training of the enumerators and (c) the daily check of all collected surveys from the research team to ensure that data were captured properly. Regarding (a) we asked the fish production-sales-self-consumption questions that were mostly sensitive to such recollection problems in the same loop for any given individual fish species in a pond. In other words, we asked for each fish species the questions for income generation and self-consumption immediately after the production questions. At the same time coded in the tablets each of these loops to help identify discrepancies if the sums for production, sales, and self-consumption did not add up. Regarding (b) we opted for in-person interviews at the house of the respondents (and not some common area) to ensure that we had the undivided attention of the interviewees and that they could consult their farm production diaries (if available and needed). Furthermore, we instructed enumerators to spend extra time on such questions and ensure that the quantities balance. Regarding (c) at the end of each day the collected data was uploaded by the data collection team and checked by the research team to ensure consistency for such data and provide timely feedback to enumerators for such sensitive variables. A comprehensive outline of the quality assurance methods adopted in the study can be found elsewhere (Gasparatos et al. 2024). Although we cannot disregard the possibility of data collection errors, our findings for such recollection-sensitive variables seem to reflect well the existing evidence from Bangladesh (Belton and Azad 2012; Jahan et al. 2015).

Third, we must acknowledge that DCE models using log normal cost coefficient can be considered as unstable (McFadden 2022). Acknowledging this, our intention here is to understand for each DCE the relative preferences across different characteristics of improved strains (with cost being one of them) within and between commercialization clusters. It is not to estimate the actual WTP for improved strains or prices. In this sense the results of the DCEs should be interpreted carefully. In particular rather than an exact estimate of WTP for fingerlings, they should be interpreted as the comparative preference for fingerling cost compared to the other attributes in each of the three DCEs.

## Results

### Production characteristics

**Fig. 1:**
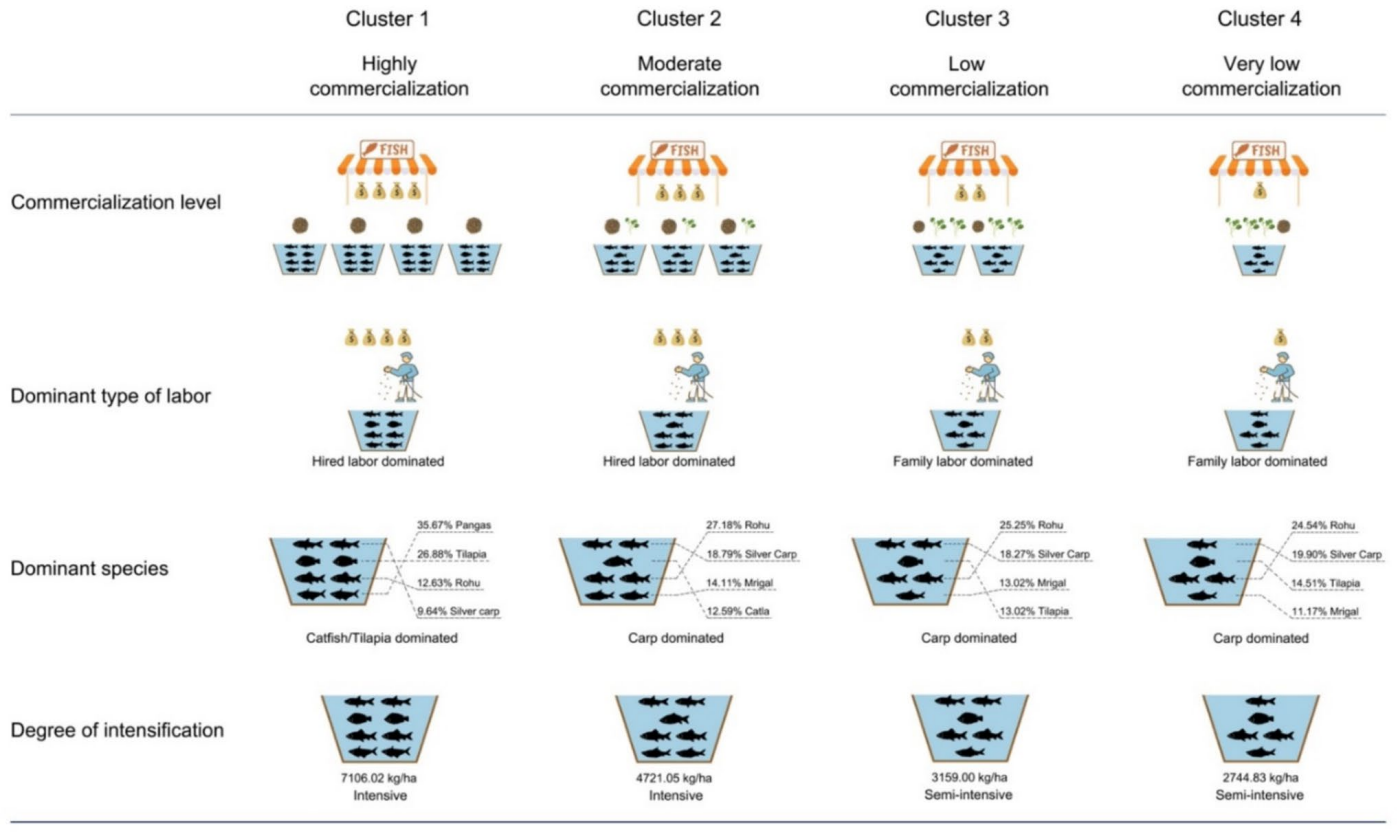
Typology of carp production systems in Bangladesh

The cluster analysis (see Methods) identifies four distinct carp production systems (Fig. 1). Figure S5-6 (Supplementary Material) contains the values of the 28 production variables considered for the clustering analysis, and the differences between the identified production systems using one-way ANOVA tests (see “Step 4: clustering analysis and typology development”). Figure S7 (Supplementary Material) contains the demographic characteristics of the producers in each system. Collectively the outcomes of the clustering and the ANOVA analyses show a clear differentiation between the four carp production systems as outlined below.

Cluster 1 is the smallest cluster and contains 8% of the producers. It is characterized by the highest commercialization rates (79.2 points; see explanation in Methods). Carp production is usually a secondary activity in this cluster, occuring mainly in conjunction with intensive tilapia or catfish production. The production systems in this cluster are characterized by the highest levels of commercial feed use (12,045 kg/ha/yr) and stocking density (11.6 thousand per ha), resulting in the highest intensification in terms of yield (7106 kg/ha/yr). This is achieved despite comparatively average pond sizes (0.58 ha) and levels of BMP adoption. The fraction of hired labour (and related cost) is quite high within this cluster.

Cluster 2 contains 31% of producers and is characterized by the second highest commercialization rate (68.5 points). Carp species production is dominant in this cluster, with the production systems characterized by the second highest levels of commercial feed use (3521 kg/ha/yr) and stocking density (8.0 thousand per ha) These result in the second highest degree of intensification in terms of yield (4721 kg/ha/yr). Production systems in this cluster are characterized by the largest pond sizes (1.16 ha), the highest BMP-adoption rates, and comparatively high dependence on hired labour (and associated costs).

Cluster 3 contains 50% of the producers. It is characterized by relatively low commercialization rates (50.7 points). Carp species production is dominant in this cluster, with the production systems characterized by relatively lower levels of commercial feed use (1530 kg/ha/yr) and stocking density (3.2 thousand per ha), compared to the more commercialised clusters above. This translates into semi-intensive production systems in terms of yields (3159 kg/ha/yr), despite average BMP adoption rates. Family labor is very prevalent (second highest among clusters), and as a result labour costs are relatively low (second lowest among clusters).

Cluster 4 contains 11% of producers. It is characterized by very low commercialization rates (39.1 points). Carp species production is dominant in this cluster, but the production systems are characterized by the lowest commercial feed use (1204 kg/ha/yr), stocking density (0.9 thousand per ha), BMP adoption and pond sizes. Furthermore, they almost completely rely on family labour. Collectively, these characteristics result in semi-intensive production systems that have the lowest yields (2745 kg/ha/yr).

Beyond these variations in production systems' characteristics, there is also significant differentiation in cultivated carp species among clusters. More commercialized clusters tend to either specialise in high-value carp species such as rohu (*Labeo rohita*) and catla (*Catla catla*) (Cluster 2), or produce them jointly with other fish species that can achieve high yields such as pangasius catfish (*Pangasius pangasius*) and/or tilapia (*Oreochromis mossambicus*) (Cluster 1) (Figure S8, Supplementary Material). Conversely, although in less commercialised clusters (Cluster 3–4) there is some production of high-value fish species, there is an overall higher prevalence of lower value carp species such as grass carp (*Ctenopharyngodon idella*), common carp (*Cyprinus carpio*), mrigal (*Cirrhinus cirrhosus*), and small indigenous fish species (Figure S8, Supplementary Material).

Finally, males almost completely dominate carp aquaculture production. Males were overwhelmingly and consistently responsible for pond operation in every cluster (> 98%), with no singificant diefference between clusters (Figure S9, Supplementary Material). Similarly, males overwhelmingly made decisions over fish sales and profit (Figure S9c-e, Supplementary Material). However, there are some noteworthy differences. In marginally more households in the low and very low commercialisaiton clusters (Cluster 3–4) women are responsible for sales and profit decisions compared to high and moderate commercialisation clusters (Cluster 1–2) (Figure S9c-e, Supplementary Material). Although the absolute numbers of these households is still very low, the differences between clusters are statistically significant.

### Sustainability performance and experience of climatic shocks

**Fig. 2:**
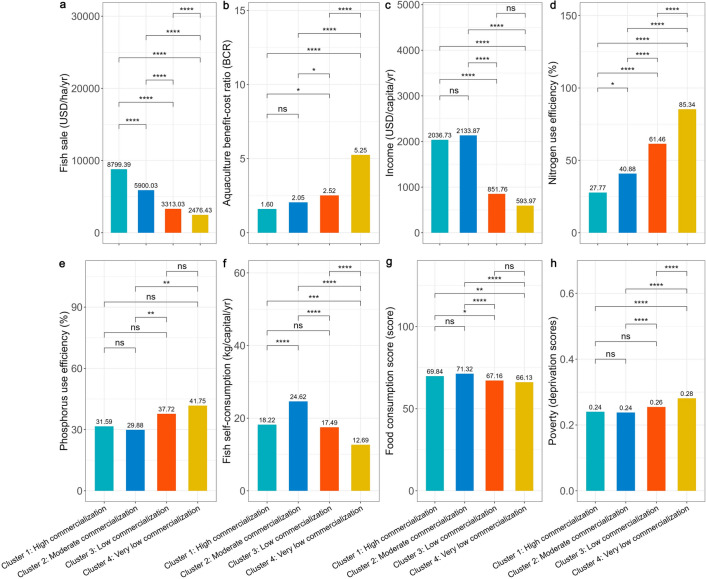
Sustainability performance of production systems in each cluster. Numbers above bars denote mean scores for each sustainability performance indicator for each cluster. The horizontal square brackets represent the pairwise comparisons between the cluster at the opposite tips of the brackets. Symbols above each square bracket denote the statistical significance of the differences as follows: ‘*’ *P* < 0.05, ‘**’ *P* < 0.01, ‘***’ *P* < 0.001, '****' *P* < 0.0001, and ‘ns’ not significant

Figure 2 outlines the socioeconomic and environmental performance of the production systems for each cluster. Overall, there is very clear differentiation in sustainability performance considering the statistically significant differences for all indicators across almost all pairwise comparisons.

In terms of economic indicators, there is significant differentiation in fish-related income, with producers in Cluster 1 having the highest average incomes and producers in Cluster 4 the lowest (Fig. 2a). This is to be expected considering that commercialization is a major distinguishing factor between production systems across clusters (Fig. 1). There are also significant differences in total household income, with more commercialized producers (Cluster 1–2) having significantly higher incomes compared to less commercialized households (Cluster 3–4) (Fig. 2c). The only non-significant differences in total income are between Clusters 1 and 2, and between Clusters 3 and 4 (Fig. 2c). Conversely, producers with very low commercialization (Cluster 4) have the highest BCR, followed by producers with low, moderate and high commercialization (Cluster 3, 2 and 1 respectively) (Fig. 2b). This is due to the fact that although lower commercialization groups have lower fish income (see above), they also have much lower aquaculture production costs. Aquaculture production costs are mostly associated with feed and labour costs, which are very low for Clusters 3 and 4 that generally depend on low commercial feed input and family labour (see previous section). The differences in BCR are statistically significant for all pairwise comparisons, with the exception of high and moderate commercialized groups (Fig. 2b).

In terms of environmental indicators, we observe a significant increase in nitrogen use efficiency across commercialization levels (Fig. 2d) Less commercialized clusters (Cluster 3-4) have higher nitrogen use efficiency,with the differences being statistically significant for all pairwise group comparisons. However, there is a less clear increase in phosphorus use efficiency (Fig. 2e), with statistically significant differences for only two pairwise comparisons.

In terms of social indicators, Cluster 2 (Moderate commercialization) has both the highest fish self-consumption and dietary diversity (measured as Food Consumption Score, see Methods) compared to other clusters (Fig. 2f, g). Low and very low commercialization clusters (Cluster 3 and 4, respectively) have consistently the lowest levels for these two indicators, with the differences being mostly significant compared to higher commercialization groups. Furthermore, there are growing poverty levels (measured as deprivation scores; see Methods) with lower levels of commercialization  (Fig. 2h). Although differences in the poverty levels of more commercialized clusters are not significant (Fig. 2g), less commercialized clusters tend to be significantly poorer.

**Fig. 3:**
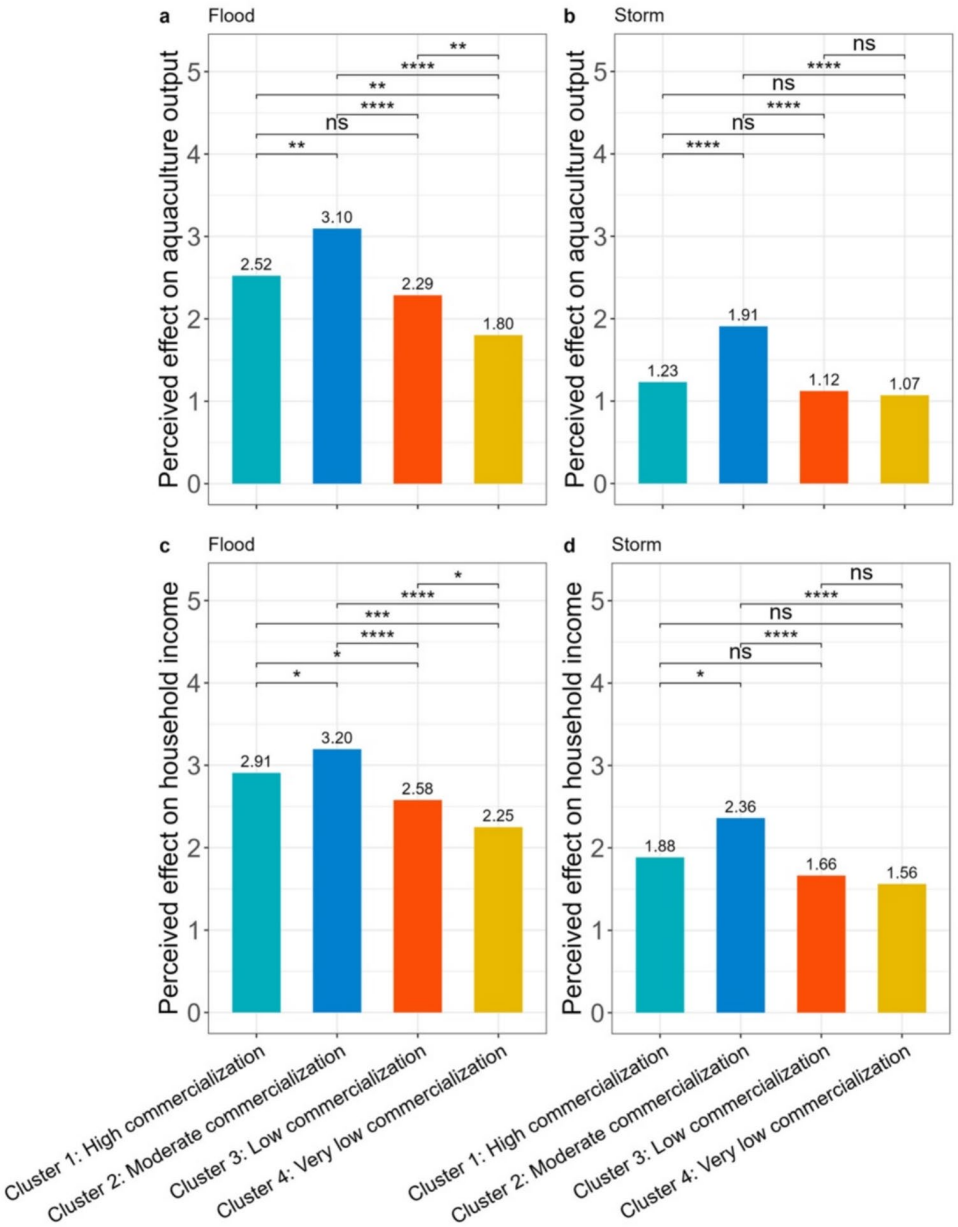
Perceived effects of floods and storms in each cluster. Numbers above bars denote mean scores for each perceived effect for each cluster. The horizontal square brackets represent the pairwise comparisons between the clusters at the opposite tips of the brackets. Symbols above each square bracket denote the statistical significance of the differences as follows: ‘*’ *P* < 0.05, ‘**’ *P* < 0.01, ‘***’ *P* < 0.001, '****' *P* < 0.0001, and ‘ns’ not significant

Finally, many of the study households experienced at least one type of climatic shock in the 5 years prior to the survey (Table S19, Supplementary Material). Floods and storms were the most commonly experienced climatic shocks, both across individual clusters and the entire sample. Figure 3 indicates significant differences in the perceived effects of floods and storms on aquaculture output and household income. Overall, floods and storms had significantly higher perceived negative effects on aquaculture output and household income in more commercialized clusters (Cluster 1—2) compared to less commercialised clusters (Cluster 3 and 4). Notably, the perceived negative effects of floods on household income and aquaculture output were reportedly more severe than the perceived effects of storms across all lusters.

### Preferences for attributes of improved fish strains

We employed three DCEs to elicit preferences for the attributes of improved fish strains. Each choice experiment had a different theme, such as productivity performance (DCE1), fish traits (DCE2) and pond support systems and management (DCE3) (see “Methods” and Table S16-S18 in Supplementary Material).

**Fig. 4:**
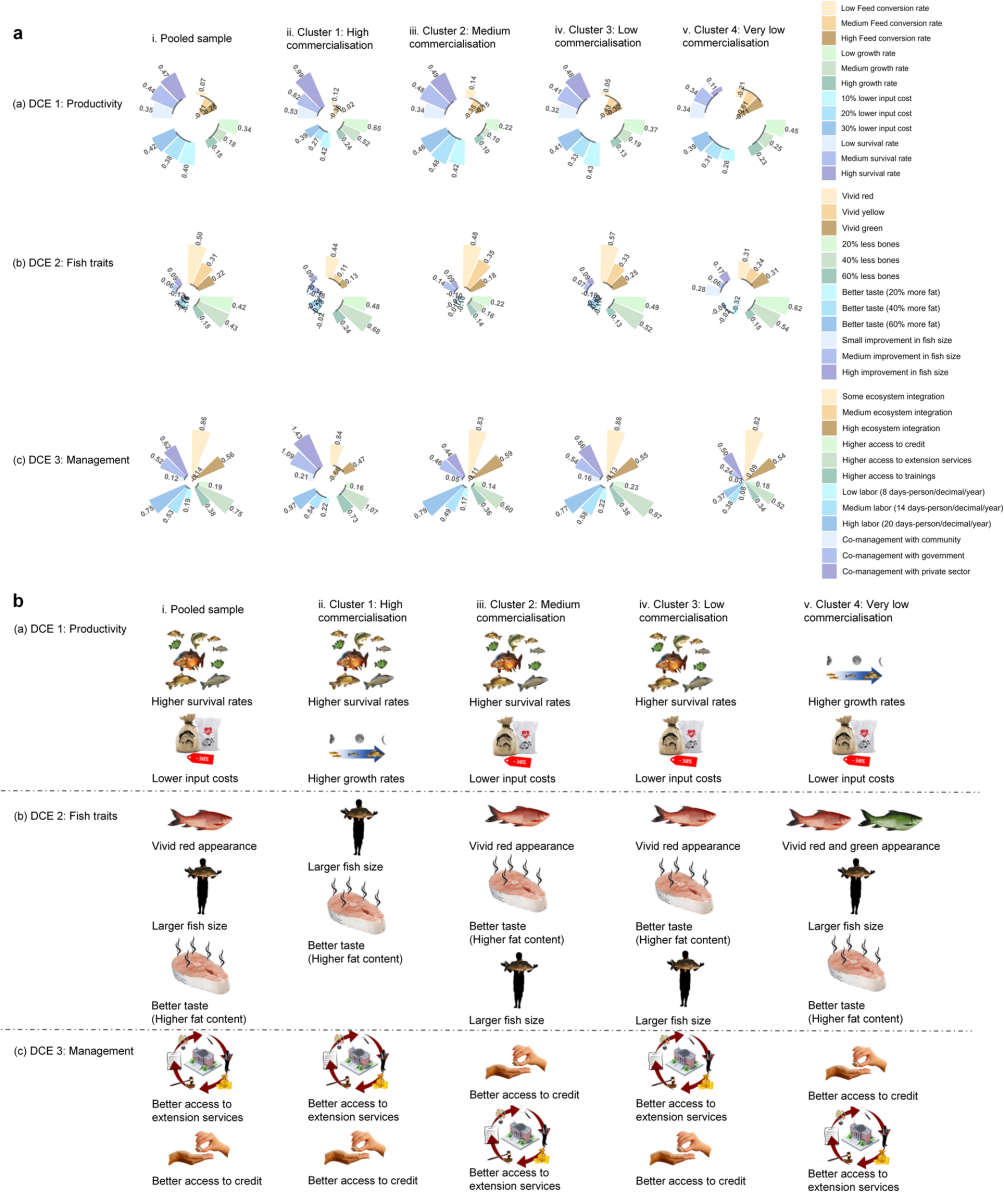
Preferences for the attributes of improved fish strains. Panel **a** depicts the estimates from the choice experiment, with each circular bar chart containing the preferences for the different attributes and levels for the pooled sample (column i) and individual clusters (columns ii–v). Each circular bar chart consists of four sectors marked in different colors that denote the individual attributes. Each of these distinct sectors contains three bars that denote the levels of preferences for the specific attribute. Longer bars and higher numbers denote higher preferences. Panel **b** summarizes the preferences for improved strains for each of the choice experiment for the pooled sample (column i) and individual clusters (columns ii–v)

Overall, all clusters have positive preferences for improved productivity, fish traits and management practices, except for lower labor intensity and higher FCR (Fig. 4a). In particular, despite some differences between clusters, producers across all clusters generally show preferences for higher survival rates, lower input costs, larger fish size, better taste (in terms of higher fat content), vivid red appearance, and better access to extension services and credit (Fig. 4b).

Regarding productivity (DCE1), highly commercialized producers (Cluster 1) tend to have higher preferences for higher survival rates and higher growth rates (Fig. 4b). Higher survival rate is prioritized by both moderate commercialization (Cluster 2; Fig. 4c) and low commercialization producers (Cluster 3; Fig. 4d), while higher growth rate is prioritized by very low commercialization producers (Cluster 4; Fig. 4e). Producers with low commercialization generally value lower input cost (Cluster 4; Fig. 4e).

Regarding fish traits (DCE2), larger fish sizes and better taste (in terms of higher fat contents) are the most preferred fish traitsby highly commercialized producers (Cluster 1; Fig. 4g). Other producers (Cluster 2, 3 and 4) tend to have higher preference for fish appearance, followed by better taste (in terms of higher fat content) and larger fish size (Fig. 4h–j).

Regarding management (DCE3), producers from all clusters attach greater importance to higher access to extension services and credit (Fig. 4l–o). Conversely, producers from all clusters are less interested in higher ecosystem integration, and even had negative preference for labor intensity compared to other management characteristics (Fig. 4l–o).

## Discussion

### Synthesis of findings

In recent decades, inland aquaculture expanded rapidly (particularly in low- and middle-income countries of Asia), playing a crucial role in meeting nutritional needs, enhancing food security, and supporting rural livelihoods (FAO 2024). Polyculture systems of diverse carp species and other fish species operated the household and small/medium-scale business levels have been (and are still) driving much of this global expansion (Naylor et al. 2021). However, this expansion has been uncoordinatedand characterized by fragmented investments and sporadic efforts to adopt improved production practices (Wang et al. 2023). Furthemore, the sustainability of these efforts has often been criticsed.

Importantly for this paper, at the route of these problems is partly the assumption of a certain degree of homogeneity in the characteristics, performance and needs of small-scale aquaculture producers (Short et al. 2021). An underlying reason is the lack of high-quality benchmark information about the characteristics and sustainability perfornace of such systems, and the preferences of producers.

#### Characteristics and perfomance

We identified four distinct small-scale carp aquaculture production systems in Bangladesh. The most intensive and commercialised (Cluster 1–2) are characterized by higher stocking densities and reliance on commercial feed and hired labour (Fig. 1, Figure S5-S6, Supplementary Material). Furthermore they specialise either in high-value carps (Cluster 2), or jointly produce them with other fish species such as tilapia or catfish (Cluster 1), which though considered to be rather low-value species they could be cultured at high densities (and thus achieve higher productivity compared to other species). Due to the generally lower price, tilapia and pangasius have high market demand among poorer consumers in Bangladesh. These translate in the stronger market orientation, higher yields, better social and economic performance and generally lower environmental performance of production systems in these clusters (Figs. 1 and 2), as well as the higher perceived effects of climatic shocks (Fig. 3).

Conversely, less intensive and commercialised production systems (Cluster 3–4) are characterized by lower stocking densities, lower commercial feed use, and higher reliance on family labour (Fig. 1, Figure S5-S6, Supplementary Material). Such systems mainly rely on lower value carps, often in conjuction with small indigenous fish species. Notably even when producing high value carp species, the fish are generally smaller in size due to the generally lower commercial feed inputs, and thus command lower market prices. Hence, these production systems tend to have lower market orientation, yields and socioeconomic performance, but comparatively better environmental performance (Figs. 1 and 2) and lower perceived effects of climatic shocks (Fig. 3).

When looking at our findings more critically, we can identify three important patterns between clusters, namely (a) reliance on multiple fish species, (b) performance trade-offs, and (c) gender imbalance in the operation and decision-making of carp aquaculture systems. We unpack each of these aspects below.

First, each cluster produces on average > 4 different fish species, with Cluster 2 reporting on average 5 fish species (Figure S6, Supplementary Material). This reflects the fact that polyculture systems are very prevanlent in Bangladesh and the wider region (Samanta Chandan and Roy 2024). However, our study notably points that this reliance on multiple species characterises the entire carp aquaculture sector, even the highly intensified production systems (Cluster 1–2). This highlights the importance of biological and genetic diversity within the carp aquaculture sector, which should be considered in sustainable intensification efforts (“Implications for sustainable intensification”).

Second, we observe sustainability trade-offs within clusters The more commercialised and intensified clusters (Cluster 1–2) perform significantly better for social and economic indicators than the less commercialised and intensified clusters (Cluster 3–4). Conversely, the less commercialised and intensified clusters (Cluster 3–4) generally have a better environmental performance due to the signifciantly lower use of commercial feed (Figure S5, Supplementary Material), which is associated with higher water pollution from nutrient runoff (Burkholder et al. 2007). Such trade-offs have been identified and in other aquaculture contexts in low- and middle-income countries (Avadí et al. 2015; Henriksson et al. 2018), and should be considered in sustainable intensification efforts (“Implications for sustainable intensification”).

Finally, despite the clear differentiation in terms of production characterstics and sustainability performance, there is very large homegeneity among clusters in terms of gender dynamics. Males have a disproportoinate role in pond operation and fish sales/profit decisions in all clusters, which is quite consistent with other studies in Bangladesh (e.g., Njogu et al. 2024) and other low- and middle-income countries (Kruijssen et al. 2018). This has been attributed to diverse cultural, socioeconomic and institutional factors including religion, tenure rights, and access to credit (De and Pandey 2014; Kusakabe and Thongprasert 2022), which collectivelly create barriers for the entry of women in aquaculture. Interestingly, women are slightly more responsible for fish sales/profit decisions in low commercialisation clusters (Cluster 3–4) (Figure S9c-e, Supplementary Material). This reflects studies on women leadership in the operation of aquaculture ponds and the marketing of related output in less commercialsed production models (e.g., aquaculture production in underutilized ponds) (Dam Lam et al. 2022; Kruijssen et al. 2018). This gender disparity should be tackled in sustainable intensification efforts (“Implications for sustainable intensification”).

#### Preferences

Cluster differentiation is also reflected in producers' preferences for the attributes of improved fish strains as elicited from the three choice experiments. Improved fish species are one of the possible options for the sustainable intensification of aquaculture systems (FAO 2024; Houston et al. 2020).

On the one hand there is some degree of preference homogeneity among all clusters in terms of high preference for higher fish survival rates, higher fish growth rates, larger fish size, better taste (in terms of higher fat content) and appearance (Fig. 4). These results are consistent with past surveys in Bangladesh and India about fish trait preferences for genetic improvements of rohu (*Labeo rohita*) (i.e. high preference for better growth rates, larger fish size, appearance, and taste) (Mehar et al. 2022).

On the other hand, we observed significant preference  heterogeneity, especially in relation to productivity (DCE1) and fish traits (DCE2). For instance, producers with lower levels of commercialization (Cluster 3–4) tend to rate more highly the lower input costs, compared to producers in highly commercialized cluster (Cluster 1–2). Furthermore, fish appearance was prioritized compared to other fish traits (e.g., size, taste) by producers with lower commercialization (Cluster 3–4), while producers with high commercialization  preferred larger fish size and taste (Cluster 1–2). Such variability has been observed in other studies exploring farmers’ preferences for improved seeds (Sánchez-Toledano et al. 2017) and livestock traits in different production systems (i.e., subsistence and market-oriented) (Roessler et al. 2008).

In terms of aquaculture management preferences (DCE3), producers from all clusters attached generally higher importance to better access to extension services and credit. This underlines the criticality of aquaculture extension delivery for small-scale aquaculture producers, which is strongly associated with the proper implementation of aquaculture interventions (Dompreh et al. 2023; Ragasa et al. 2022). This also reflects the fact that farms lacking adequate investment or operating capital will likely not be able to adopt innovations, including those with sustainable intensification potential. While aquaculture is capital intensive and requires sustained capital availability, access to credit can help to overcome constraints of capital availability (Kumar et al. 2018; Wang et al. 2024).

Finally, while these findings clearly reflect producers’ preferences for productivity, fish traits and aquaculture management, we recognize some analytical limitation. In particular the economic rationality assumption underlying these results (i.e. in the Random Utility Model, “Step 6: choice experiment”) views decision-makers as fully rational individuals that can consistently maximize utility based on well-defined preferences. However, farm decision-making is more complicated in reality Several studies have shown that decisions over the adoption of agricultural technologies can deviate from this assumption due to sociocultural and behavioral factors, such as sociocultural norms, cultural traditions, peer effects, social learning and cognitive heuristics (Foolen-Torgerson et al. 2023; Kassie et al. 2022; Villamayor-Tomas et al. 2019; Vortkamp and Hilker 2023). We acknowledge that although our findings were not influenced by other economic variables, these factors may shape preferences beyond monetary payoffs, influencing farmer’s decision-making processes.

### Implications for sustainable intensification

Our study responds to the calls for understanding, representing and accounting for diversity and differentiation between and within small-scale aquatic food systems (Short et al. 2021). By providing a very rich picture of the differentiated characteristics, performance and preferences of carp producers in Bangladesh it helps establish a robust benchmarkthat can guide the sustainable intensification of the aquaculture sector in the country as discussed below. This is particularly useful in the context of major national policies and plans that are either specific for the aquaculture sector (e.g. the “National Aquaculture Development Strategy and Action Plan (NADSAP) 2013” (FAO and MFL 2014)), or are cross-sectoral such as the National Adaptation Plan (MEFCC 2022). Furthermore, it can be relevant to broad economic development policies and plans  e.g., 8FYP (GED 2020a) or the vision document “Perspective Plan of Bangladesh 2041” (GED 2020b).^1^

First, it can help assess the success of future interventions in the aquaculture sector, serving as a critical baseline from which progress for sustainable intensification can be measured.  Species diversification, interventions for subsistence households, and uptake of advanced technologies (incl. improved fish species) have all been identified as important for aquaculture expansion and intensification for economic development in the country (GED 2020a, b). In this sense, this benchmark does not only quantify initial producer differentiation and conditions for possible interventions as required in these plans, but also helps set performance standards against which the effectiveness of various innovations/investments/policies or other initiatives can be evaluated. Such a benchmark is instrumental in identifying where and how interventions have led to improvements, and where further action is needed.

Second, a well-defined benchmark helps fill existing data and information gaps, thereby improving the quality and effectiveness of policy and decision-making processes. As a resource it can be particularly useful in updating the National Aquaculture Development Strategy and Action Plan (NADSAP) 2013 (FAO and MFL 2014), which is generally considered outdated and needing revision (WorldFish 2024). By providing a clearer picture of the current state of the carp aquaculture sector, this benchmark can provide a better understanding of the potential trade-offs (incl. sustainability trade-offs) involved in different approaches. Decision-makers can use this information to prioritize strategies that promote the most sustainable outcomes for inland aquaculture in Bangladesh, as well as prevent possible trade-offs.

Third, this benchmark can inform and guide aquaculture investments by highlighting areas that require the most urgent attention and influx of resources. Attracting investment is necessary for ensuring both the transformation of the sector as indicated in broad economic strategies (e.g., 8FYP, “Perspective Plan of Bangladesh 2041”) as well as achieving effective adaptation as required in the National Adaptation Plan. The large heterogeneity in the sector implies that single solutions might be impossible. Investors and funding bodies can use such information to allocate capital more efficiently, ensuring that funds are directed towards interventions that target the appropriate groups and are likely to yield the highest returns in terms of sustainability and societal benefit. Ultimately, this approach could contribute to more strategic and impactful investments.

Beyond the implications of the benchmark, we identify below some further interconnected implications of our results for policy, practice and research for the sustainable intensification of small-scale aquaculture in Bangladesh and beyond. These include, (a) reducing gender disparities through coordinated actions, (b) enabling effective adaptation, (c) combating environmental trade-offs, and (d) promoting farmer-led research.

First, we observe substantial and consistent gender disparities in all clusters. All policy frameworks outlined above have strong calls for the reduction of gender disparities Overcoming these disparities would require tackling some of the systemic barriers facing small-scale aquaculture producers, which are arguably more pronounced for women (Kruijssen et al. 2018; Kusakabe and Thongprasert 2022). These include land tenure rules, access to knowledge or financial constraints, among others (De and Pandey 2014; Kusakabe and Thongprasert 2022), and can collectively affect whether and how women engage in aquaculture, adopt good practices, commercialize harvest and generate profits (Kruijssen et al. 2018). For example, clear land tenure can encourage investment in sustainable practices and infrastructure improvements (Salazar et al. 2018) and affect productivity, efficiency and profitability (Mitra et al. 2022). However, the land system in Bangladesh is characterized by multiple barriers and constraints to the recognition of women’s tenure rights (Enokwenw et al. 2024), which go beyond the aquaculture sector. Similarly, by improving access to knowledge through improved extension services, training programs, and market intelligence it is possible to enhance productivity among small-scale female producers in Bangladesh (Dam Lam et al. 2022), as it empowers them adopt innovative practices that boost both productivity and sustainability. At the same time tailored financial products and credit schemes that consider the unique challenges of small-scale aquaculture producers could provide the necessary capital for commercialization (Hanh and Boonstra 2019). Models such as patron-client relations or saving groups could enable womens' access to credit as found in other fishing and aquaculture contexts of Asia (Kusakabe and Thongprasert 2022). These aspects should be considered in any upcoming revisions of the National Aquaculture Development Strategy and Action Plan (NADSAP) 2013 (FAO and MFL 2014), which should actively seek to mainstream gender (FAO 2024).

Second, we observe rather different experiences and perceived impacts of climatic shocks between clusters with highly commercialised clusters seemingly more vulnerable, However, due to their very different characteristics, each cluster will likely both need a different approach to adaptation and face different adaptation barriers (Gunathilaka et al. 2018). For example, producers in highly commercialised clusters will likely require more substantial funding to adapt effectively onsidering their higher technology adoption and reliance on commercial feed/seed. For these clusters, adaptation approaches such as the adoption of genetically improved fish strains might be more appropriate (Sae-Lim et al. 2017). Conversely, for lower commercialisation clusters it is low-cost adaptation options (e.g. pond-dike cropping) that might be more appropriate (Ahmed and Diana 2016). Appreciating such differences between clusters should be a pre-condition when designing appropriate adaptation portfolios for each group to ensure their effective adaptation, given that aquaculture has been mainstreamed in the National Adaptation Plan (MEFCC 2022).

Third, although we did not perform a comprehensive environmental sustainability impact assessment, we observe divergences in the environmental performance of the clusters. Highly commercialised clusters (Cluster 1–2) tend to have significantly lower nitrogen and phosphorous use efficiency compared to low commercialisation clusters (Cluster 3–4). This implies their likely higher negative impact on aquatic ecosystems and biodiversity through eutrophication (Henriksson et al. 2018) and should ideally be targeted for the adoption of BMPs that reduce nutrient loading (e.g. feed management, stocking and water management) (Jahan et al. 2015; Wang et al. 2023). Beyond that more extensive analysis on their GHG emissions and land use should be performed to better understand the environmental impact of each cluster and how they can be mitigated.

Fourth, as our aim here was to obtain an accurate snapshot of the carp aquaculture sector, we followed an expert-driven approach with limited farmer involvement. However, our results strongly imply that due to their different characteristics, performance and preferences, the different clusters will likely require different interventions to achieve sustainable intensification. Future research should actively attempt to understand what these interventions should be, heavily relying on participatory processes that engage farmers from each clusters. These participatory processes should shed more light on the aspirations, needs, and capabilities of each group in achieving sustainable intensification. Furthermore, future studies should incorporate sociocultural and behavioral factors into choice experiments to develop a more realistic framework for understanding decision-making and improving policy design for sustainable aquaculture practices (Dessart et al. 2019). This knowledge can be used to develop more targeted interventions and support packages that are a better fit to the characteristics and preferences of each cluster.

## Conclusions

This study elucidated the large heterogeneity among small-scale aquaculture producers through one of the most extensive characterization studies of inland aquaculture systems up to date. We focused on carp aquaculture given its importance in many low- and middle-income countries. Using a sample of 4540 carp producers from around Bangladesh our results show the clear differentiation between carp production systems, identifying four distinct production systems across a gradient of intenification and commercialisation. The most intesified and commercialised systems (Cluster 1–2) generally have higher economic and social performance and lower environmental performance compared to less intensified and commercialised systems (Clusters 3-4). Furthermore, the different clusters exhibit some differentiation in their preferences for the attributes improved fish species, which is a promising avenue for sustainable aquaculture intensification.  Collectively, our results provide a much more nuanced picture of the carp aquaculture sector in Bangladesh, which moves beyond simple binaries (e.g., commercial vs. subsistence; intensive vs. extensive; large-scale vs. small-scale). These findings can inform interventions, investments and policies that promote improved technologies, innovations and product development (especially improved strains), as a means of achieving sustainable inland aquaculture intensification in Bangladesh, and other similar low- and middle-income countries.

## Supplementary Information

Below is the link to the electronic supplementary material.

Supplementary file1 (DOCX 7419 KB)

## Data Availability

The dataset, questionnaire and study protocol will be made available upon acceptance of the paper at: 10.7910/DVN/R01ZTM. The code will be made available upon reasonable request.

## Footnotes

1 As of the writing of this paper (April 2025), the current (interim) and future governments of Bangladesh might revise significantly all major economic plans and vision statements made by the previous government, and specifically the follow-up to the 8th FYP (GED 2020a) and vision document “Perspective Plan of Bangladesh 2041” (GED 2020b). Thus, care should be exercised when interpreting the related policy implications and recommendations.
