# Supplementary material for: Differentiated characteristics, sustainability performance and preferences among small-scale aquaculture producers: implications for sustainable intensification
Source: Sustain Sci. 2025 Aug 1;21(1):325–46. doi: 10.1007/s11625-025-01703-w (PMC12819504; doi:10.1007/s11625-025-01703-w)
Supplement: Supplementary file 1 — Supplementary file1 (DOCX 7419 KB) [file 11625_2025_1703_MOESM1_ESM.docx]

**Supporting Information for**

Differentiated characteristics, performance and preferences among small-scale aquaculture producers

Eric Brako Dompreh*, Quanli Wang*, Jie Su, Rodolfo Dam Lam, Benoy Kumar Barman, Cristiano M Rossignoli, Alexandros Gasparatos*

* Corresponding Authors

Eric Brako Dompreh **Email:** [e.brakodompreh@cgiar.org](mailto:e.brakodompreh@cgiar.org)

Quanli Wang **Email:** wang.quanli@outlook.com

Alexandros Gasparatos **Email:** [gasparatos.alex@gmail.com](mailto:gasparatos.alex@gmail.com)

**This PDF file includes:**

Supporting text

Figures S1 to S17

Tables S1 to S18

SI References

| **Box S1: Research approach for the aquaculture suitability analysis**  Step 1 entailed the development of the conceptual framework for the suitability analysis. Based on a literature review we identified three main dimensions that affect the suitability of an area for aquaculture, namely water quality, soil quality and infrastructure conditions (Ghobadi et al., 2021; Hossain et al., 2009; Nayak et al., 2018) (Table S2). Based on this review we also selected 10 specific indicators that span these dimensions (a) phosphate content (in mg/l), (b) water hardness (in mg/l), (c) soil pH, (d) soil texture (in % clay content), (e) soil organic matter (in % of carbon content), (f) distance to water sources (in m), (g) distance to road (in m), (h) elevation (in m), (i) slope (in %), and (j) population density (in persons/km^2^). Based on the literature, we also Following other aquaculture suitability studies in the literature (Berg et al., 2021; Falconer et al., 2016; Ghobadi et al., 2021; Hossain et al., 2009; Jayanthi et al., 2020; Nayak et al., 2018), we identify ranges and cut-off points that indicate whether a location has no suitability, moderate suitability, or high suitability for aquaculture (Table S2). We designated land use types such as built-up land and forest/conservation areas as constraints for aquaculture operations.  Step 2 entailed the collection of spatially-explicit secondary data for each of these indicators (Fig. S11) through multiple sources (Table S2). Specially, water quality data including phosphate and hardness were extracted from the Bangladesh National Drinking Water Quality Survey (Johnston, 2011). Soil quality data such as soil pH, soil texture, and organic matter content were sourced the from Harmonized World Soil Database (WIEDER, 2014). Infrastructure-related information such as water and road distributions were captured from The Humanitarian Data Exchange datasets (“Bangladesh - Contour Lines,” 2018; “Bangladesh - Rivers,” 2018; “Bangladesh - Roads,” 2018) and population density from WorldPop project (“Bangladesh - Population density,” 2015). Land use data for the constraints were obtained from a global study (Gong et al., 2019).  Step 3 entailed the harmonization of all datasets into 30×30 m pixels using ArcGIS 10.3. In more detail, individual layers were created for each of the ten indicators, and then resampled all indicator maps into maps with 30×30 m resolution, georeferenced with the projection system for WGS 1984 Transverse Mercator (Figure S12). Considering that each indicator is measured in different units, we reclassified in each of these pixels the original indicator values. For this reclassification process we use values of 1 to indicate “no suitability”, values of 2 for “moderate suitability”, and valued of 3 for “high suitability”  Step 4 entailed the development of final map of aquaculture suitability map that combines information for all indicators and provides as spatially explicit representation of aquaculture suitability in the country (Figure S14). This is achieved through the use of weighted sum overlay to combines the values of all ten indicators (Figure S15a). We assign equal weights for each indicator/layer, multiplying the reclassified values for each pixel by 0.1 and then added the values of each pixel to create the output raster (Figure S15b). We immediately exclude built up and forest/conservation areas as they were designated as aquaculture constraints (Figure S13). We round up or down to the closest whole number (Figure 15b). |
| --- |


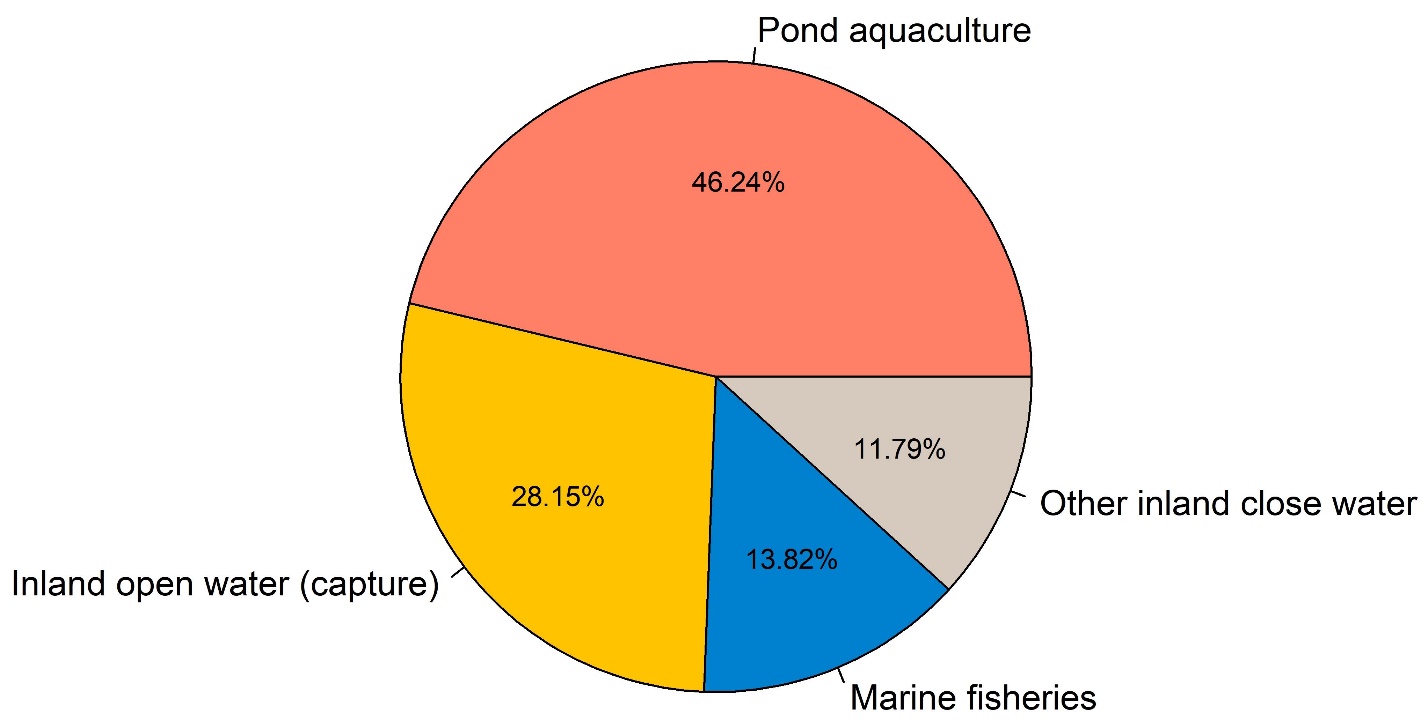


**Fig. S1.** Proportion of fish production from different inland and marine sources in Bangladesh for the 2022-23 season. Source: data extracted from Yearbook of Fisheries Statistics of Bangladesh, 2022-23 (2023).


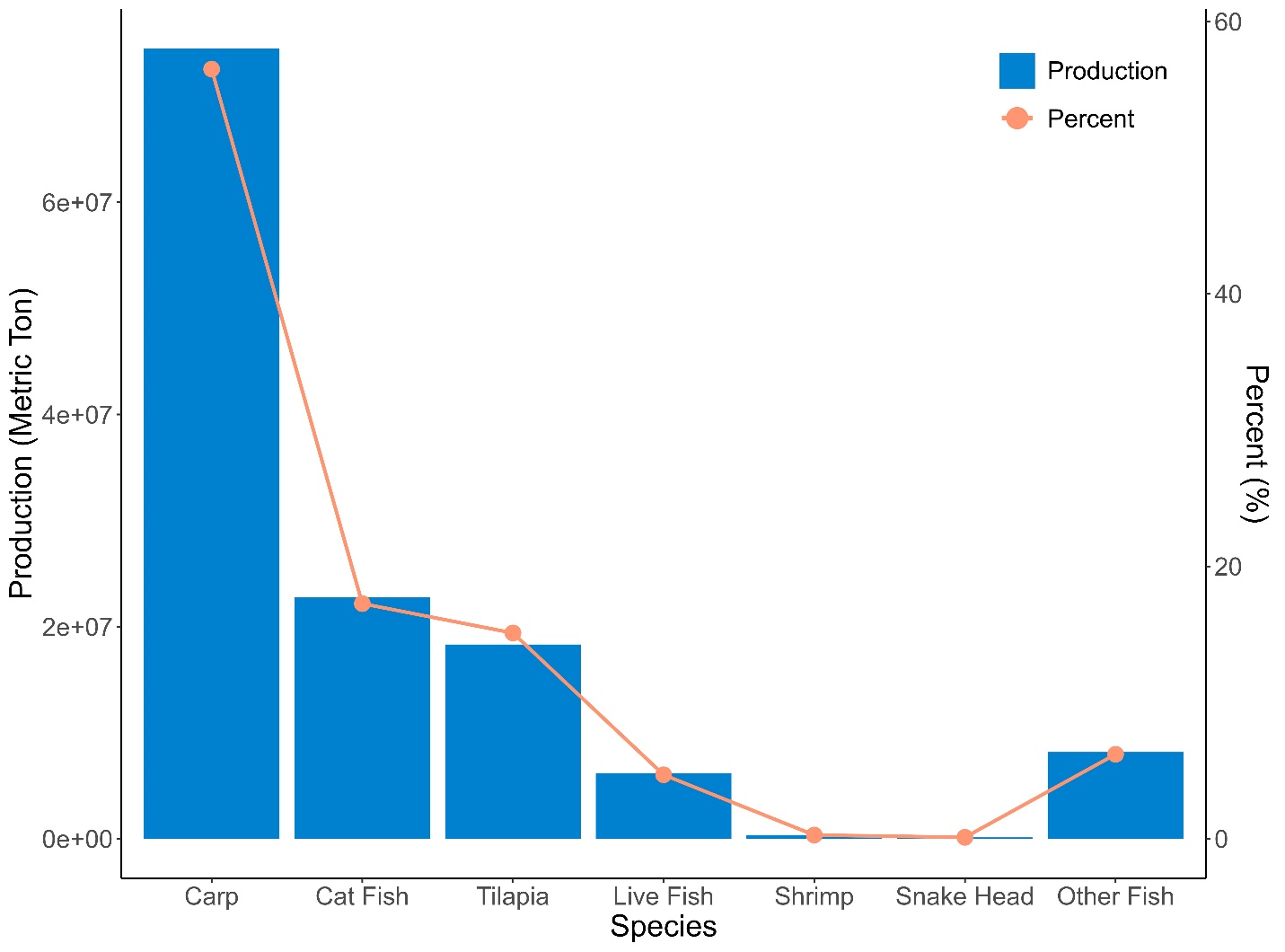


**Fig. S2.** Annual fish production in pond aquaculture by species (in tonnes) and fraction of total production by species (in %) in Bangladesh for the 2022-23 season. Source: data extracted from Yearbook of Fisheries Statistics of Bangladesh, 2022-23 (2023). Carp species includes Rohu (*Labeo rohita*), Catla (*Catla catla*), Mrigal (*Cirrhinus cirrhosus*), Kalibaus (*Labeo calbasu*), Bata (*Labeo bata*), Ghania (*Labeo gonius*), Silver Carp (*Hypophthalmichthys molitrix*), Grass Carp (*Ctenopharyngodon idella*), Common Carp (*Cyprinus carpio*), and Other Exotic Carp. Catfish species includes Pangas (*Pangasius pangasius*). Tilapia species includes Mozambique tilapia (*Oreochromis mossambicus*) and Nile tilapia (*O. niloticus*). Live fish species includes Koi (*Anabas testudineus*), Shingi/Magur (*Heteropneustes fossilis/Clarias batrachus*) and Cuchia (*Monopterus cuchia*). Shrimp includes Big Shrimp/Prawn and Small Shrimp/ Prawn. Snakehead species include Shol (*Channa striatus*), Gazar (*C. marulius*), and Taki (*C. punctatus*). Other fish species includes Sarpunti (*Puntius sarana*), silver barb (*Puntius gonionotus*) and other fish species.


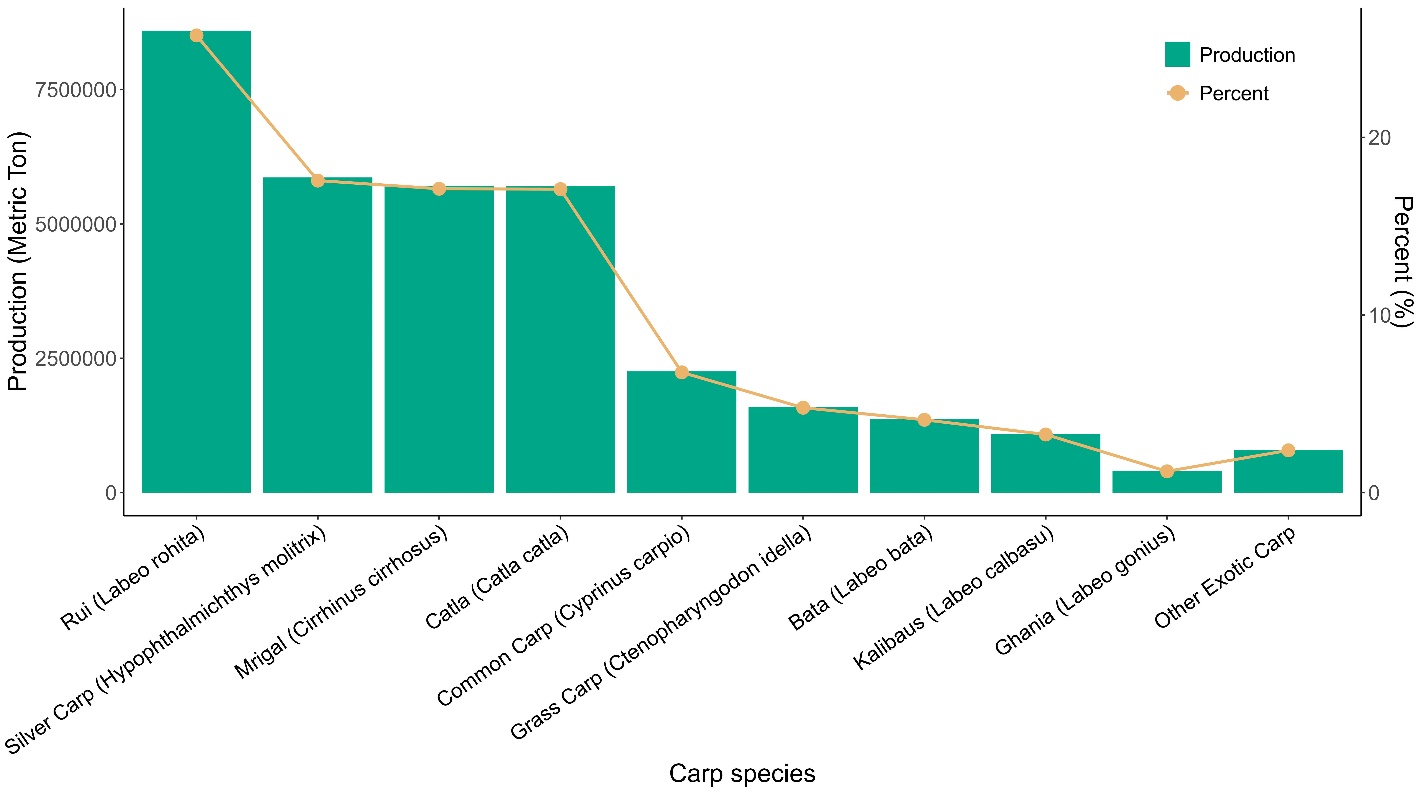


**Fig. S3.** Annual production of carp species (in tonnes) in pond aquaculture and fraction (in %) in Bangladesh for the 2022-23 season. Source: data extracted from Yearbook of Fisheries Statistics of Bangladesh, 2022-23 (2023).


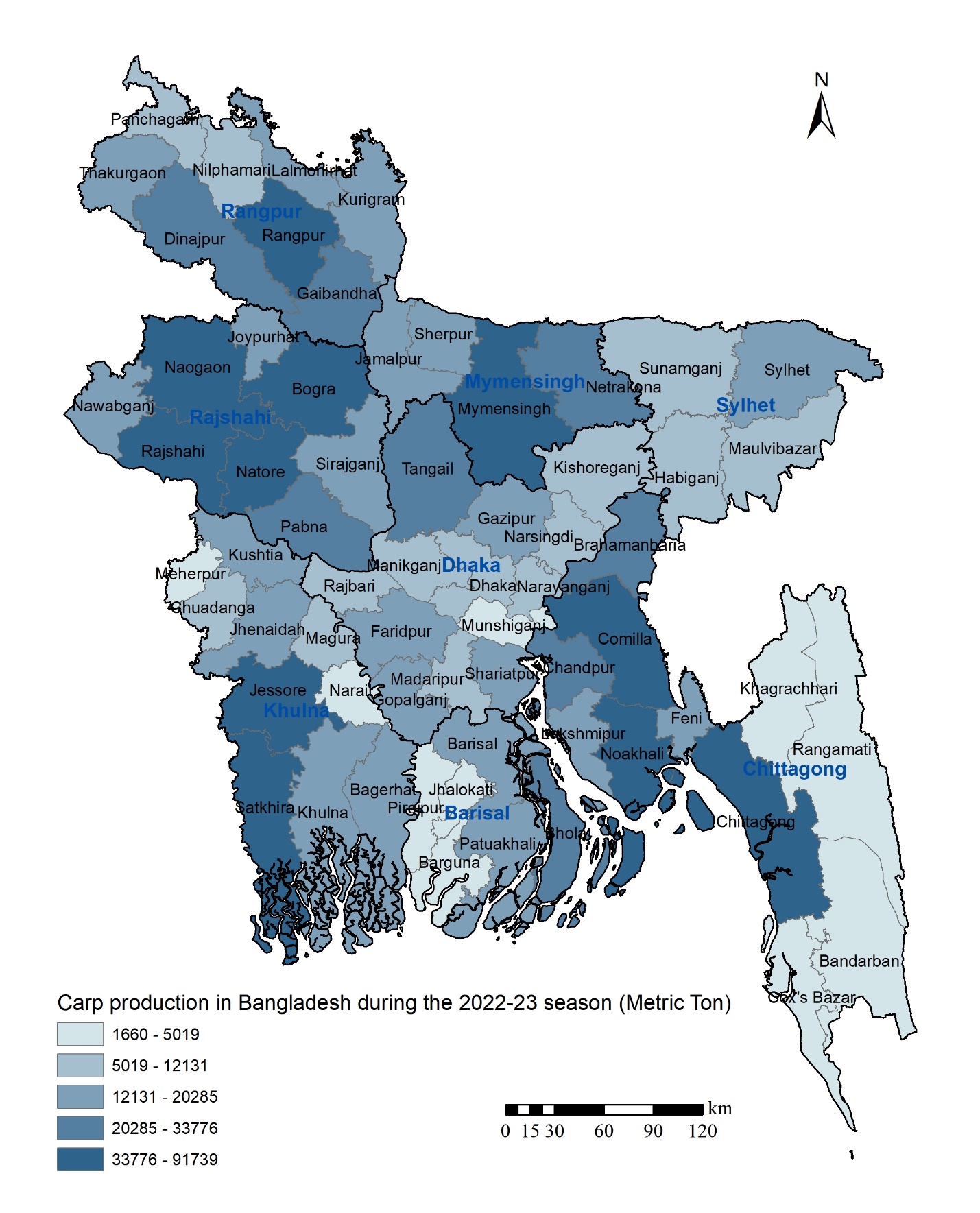


**Fig. S4**. District-level annual carp production in pond aquaculture in Bangladesh for the 2022-23 season. Source: data extracted from the Yearbook of Fisheries Statistics of Bangladesh, 2022-23 (2023).


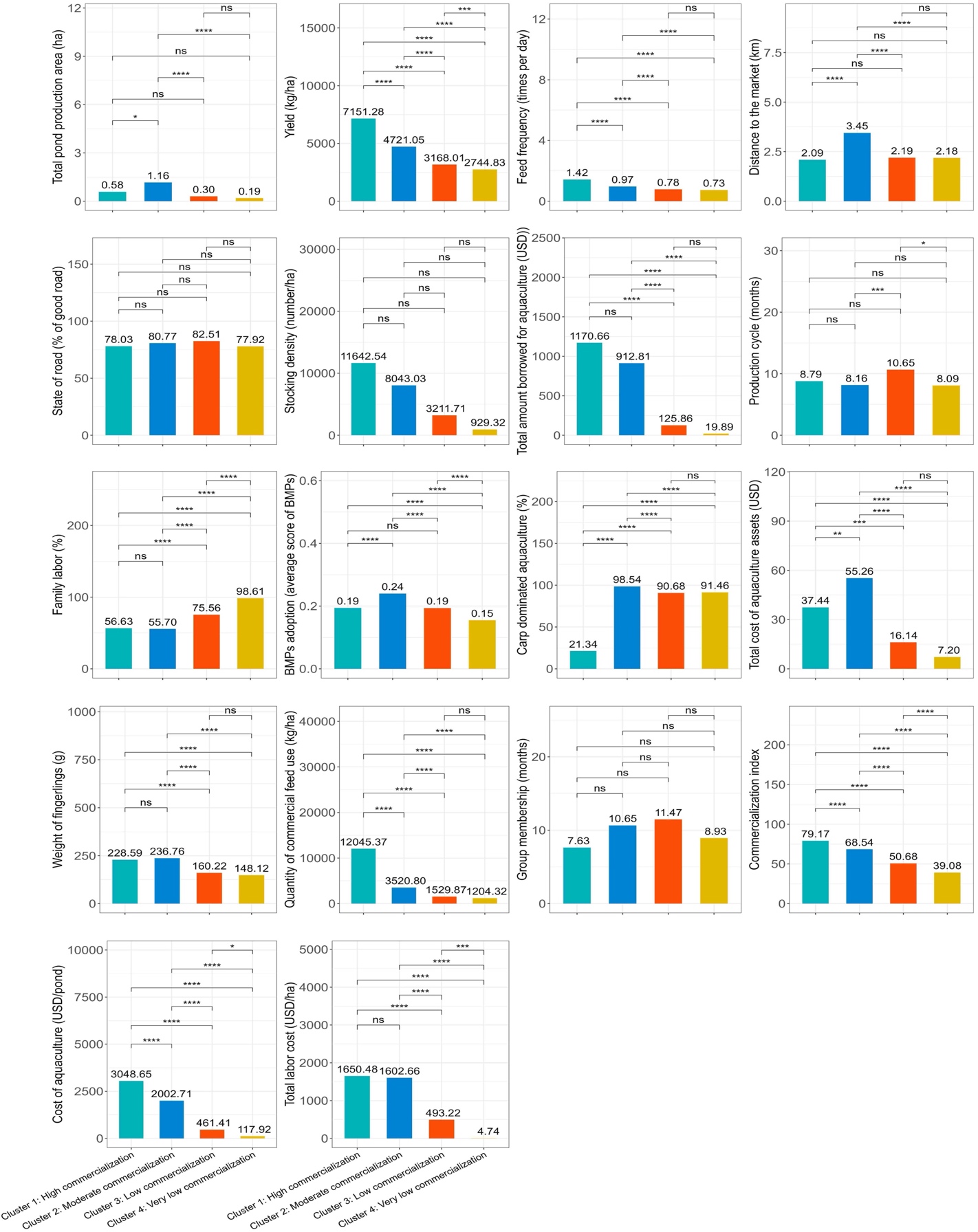


**Fig. S5**. Differences in levels of the variables used for the clustering between groups. Numbers above bars denote the mean score for each variable for each group. The horizontal square brackets represent the pairwise comparisons between the production models at the opposite tips of the brackets. Symbols above each square bracket denote the statistical significance of the differences as follows: ‘*’ P < 0.05, ‘**’ P < 0.01, ‘***’ P < 0.001, and ‘ns’ not significant.


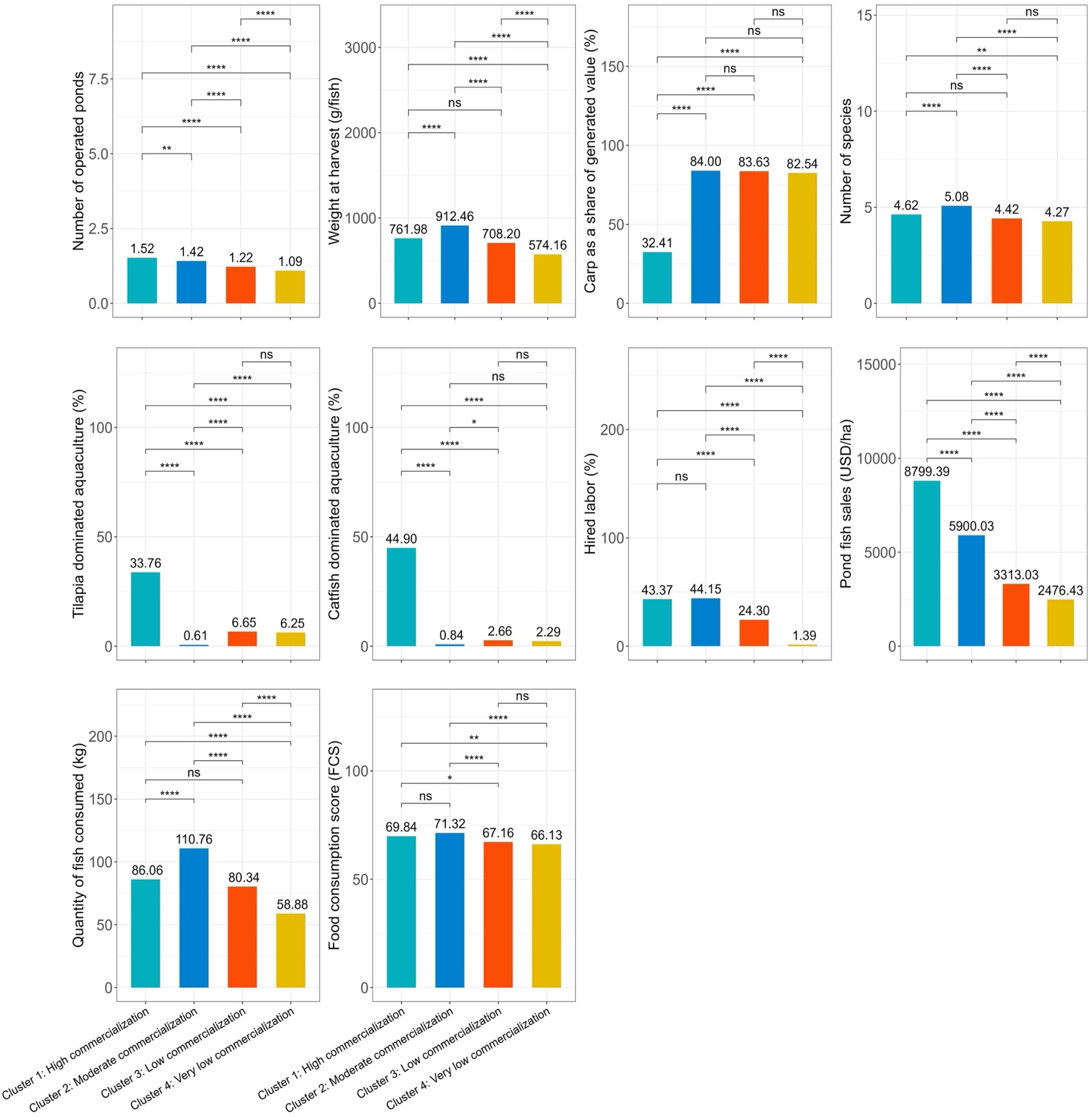


**Fig. S6**. Differences in levels of the variables not used for the clustering between groups. Numbers above bars denote the mean score for each variable for each group. The horizontal square brackets represent the pairwise comparisons between the production models at the opposite tips of the brackets. Symbols above each square bracket denote the statistical significance of the differences as follows: ‘*’ P < 0.05, ‘**’ P < 0.01, ‘***’ P < 0.001, and ‘ns’ not significant.


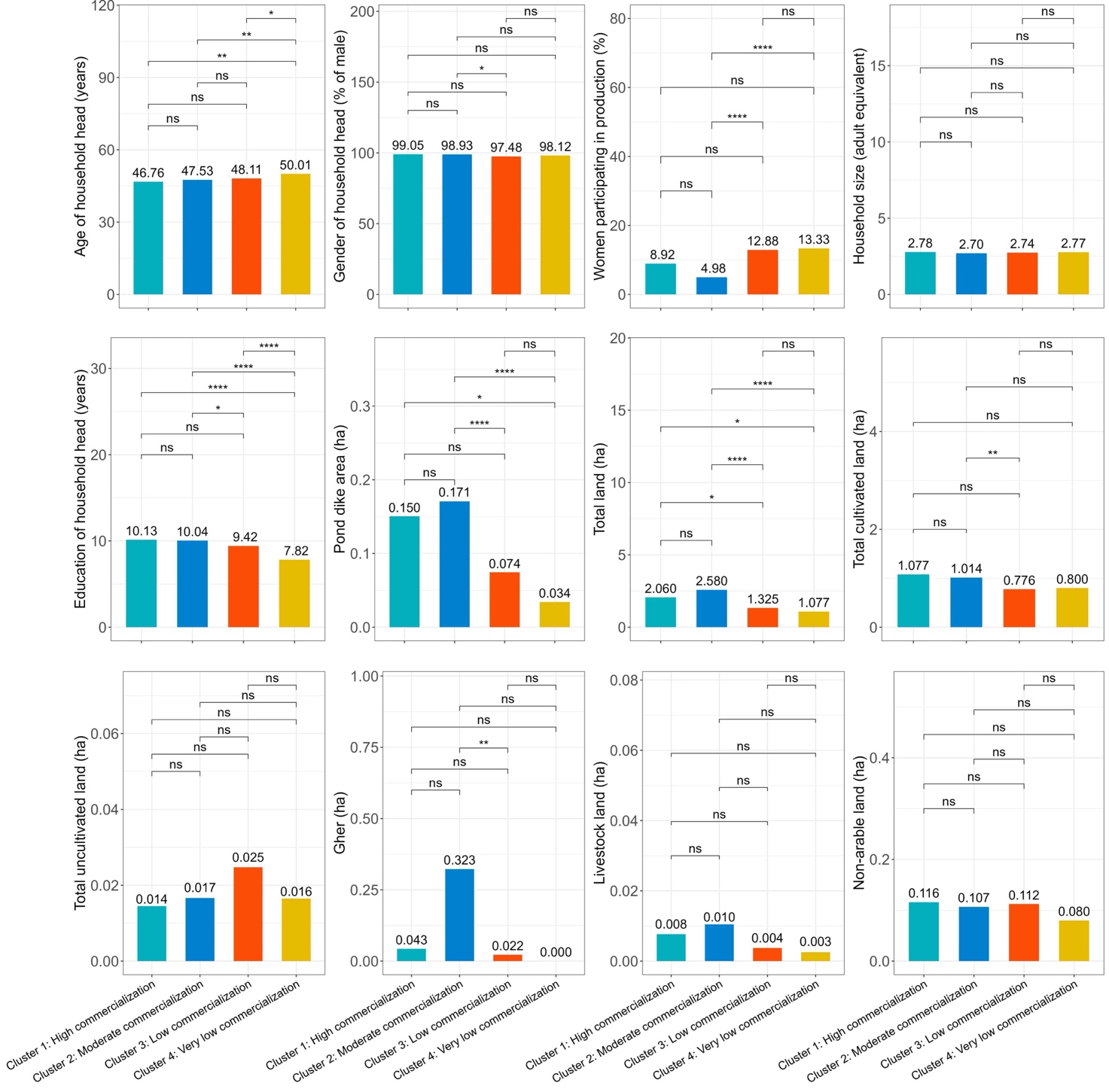


**Fig. S7.** Differences in household characteristics between groups. Numbers above bars denote the mean score for each variable for each group. The horizontal square brackets represent the pairwise comparisons between the production models at the opposite tips of the brackets. Symbols above each square bracket denote the statistical significance of the differences as follows: ‘*’ P < 0.05, ‘**’ P < 0.01, ‘***’ P < 0.001, and ‘ns’ not significant.


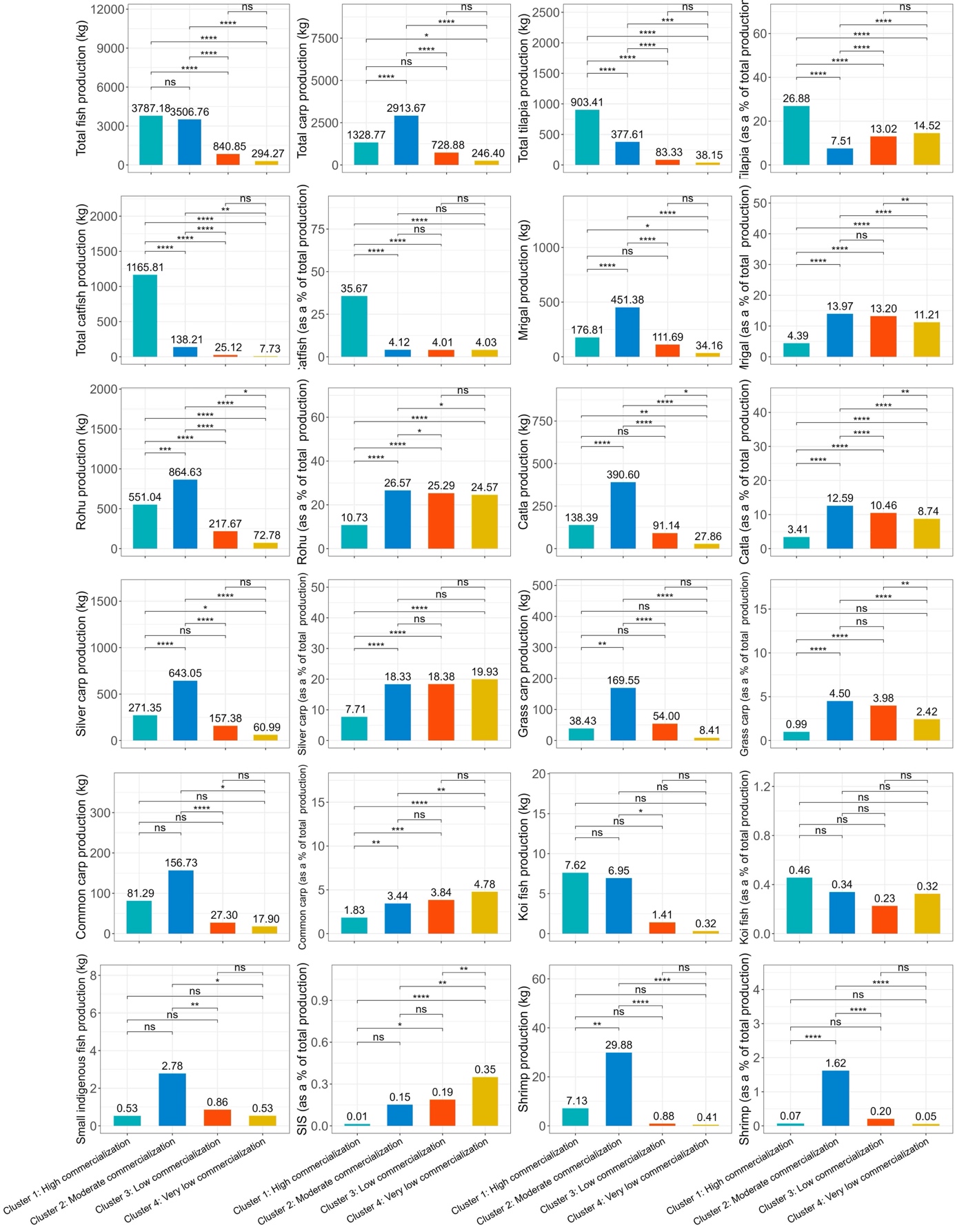


**Fig. S8.** Differences in levels of fish species production between clusters. Numbers above bars denote the mean score for each variable for each group. The horizontal square brackets represent the pairwise comparisons between the production models at the opposite tips of the brackets. Symbols above each square bracket denote the statistical significance of the differences as follows: ‘*’ P < 0.05, ‘**’ P < 0.01, ‘***’ P < 0.001, and ‘ns’ not significant.


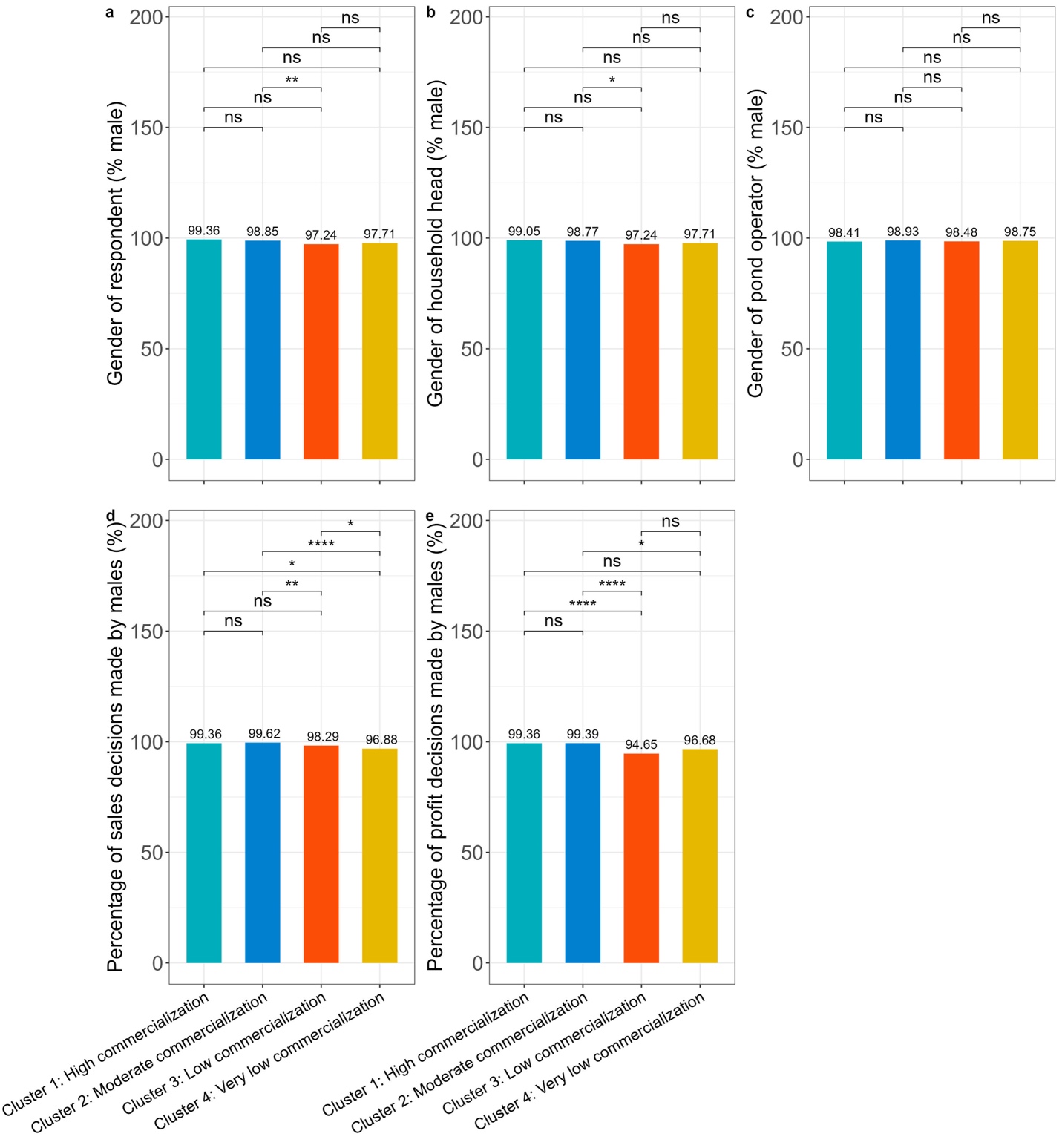


**Fig. S9.** Gender differences across the identified clusters. ‘*’ P < 0.05, ‘**’ P < 0.01, ‘***’ P < 0.001, and ‘ns’ not significant.


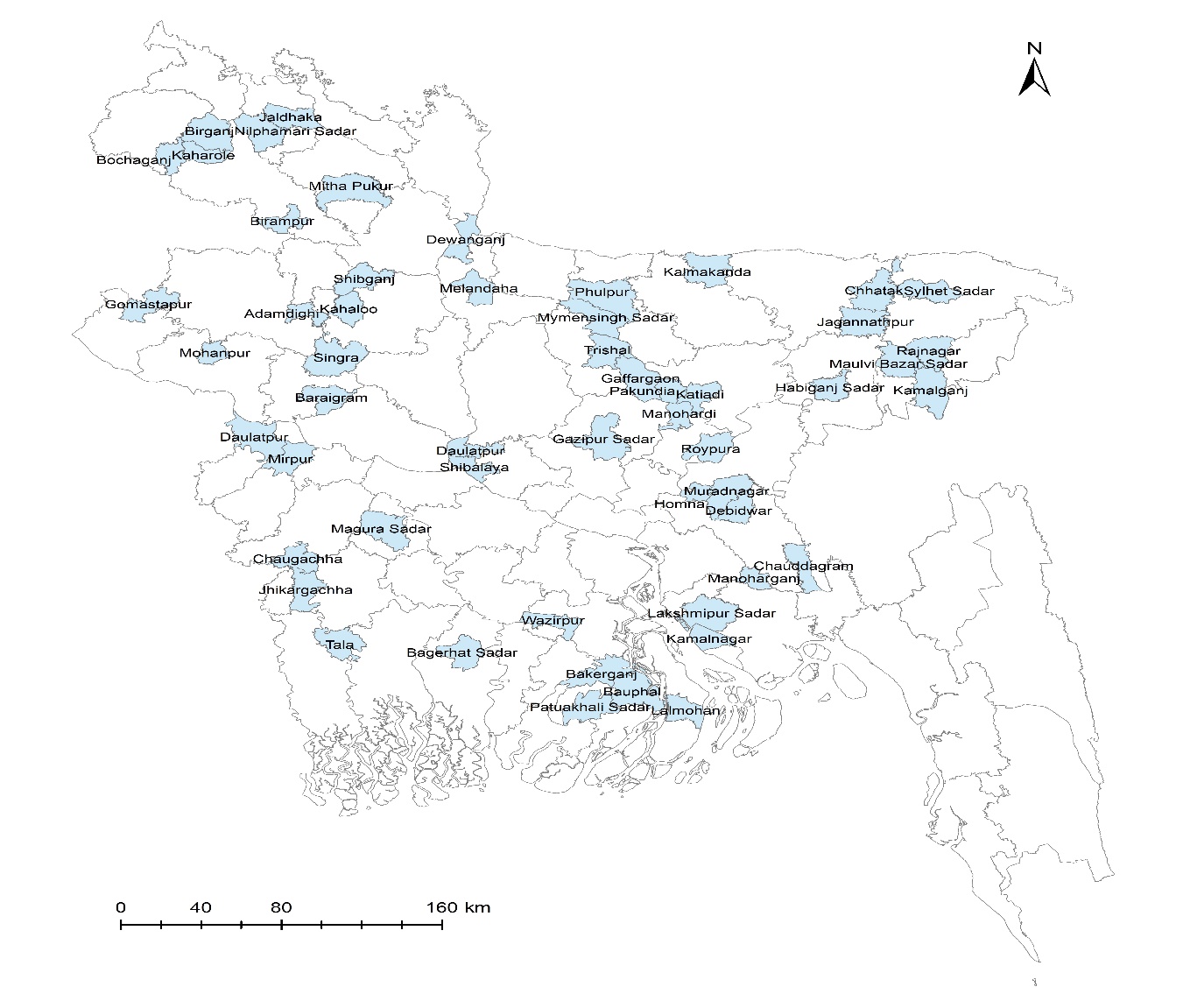


**Fig. S10.** Study upazilas.


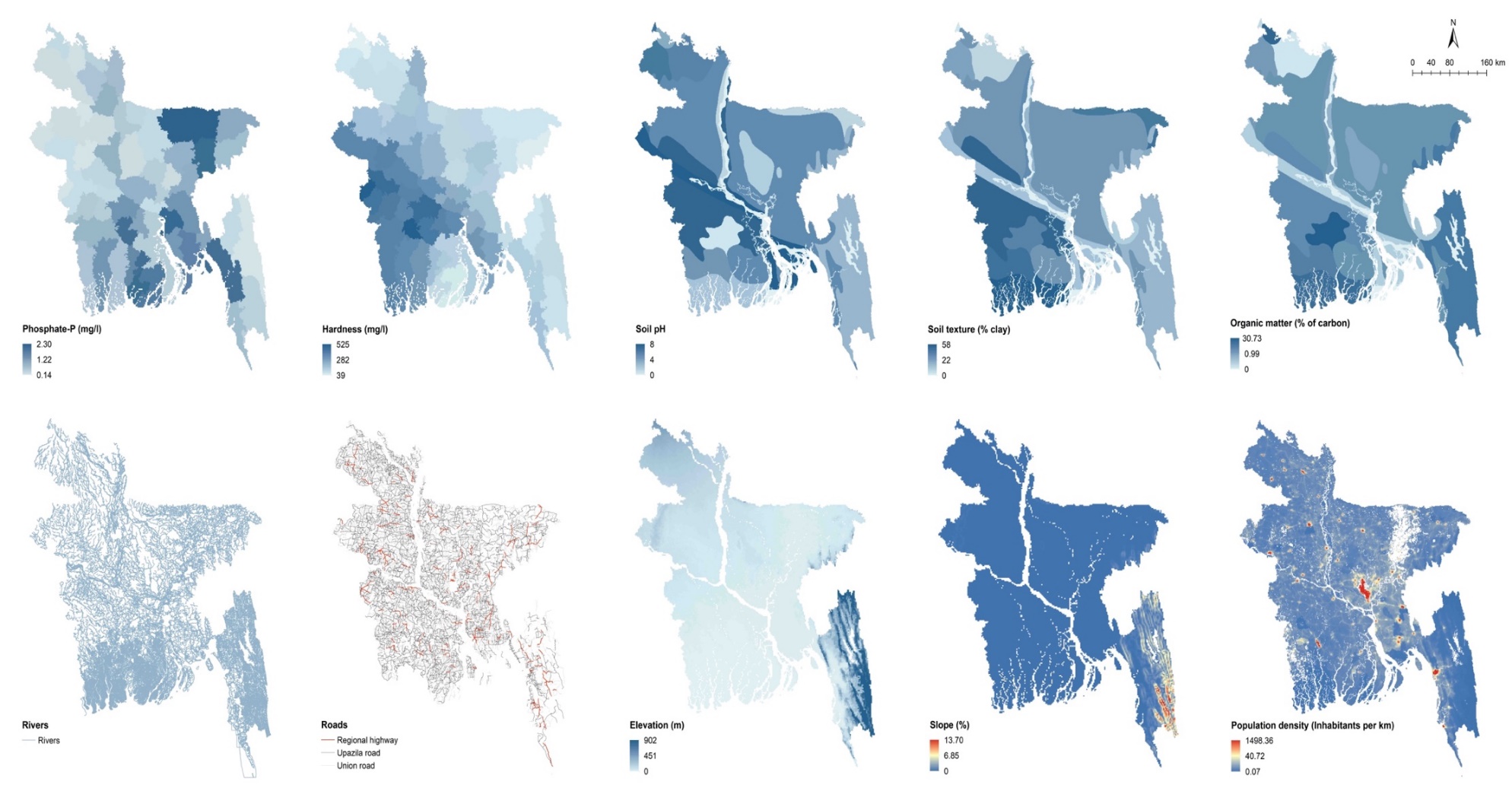


**Fig. S11.** Spatial distribution of data for the ten suitability analysis dimensions


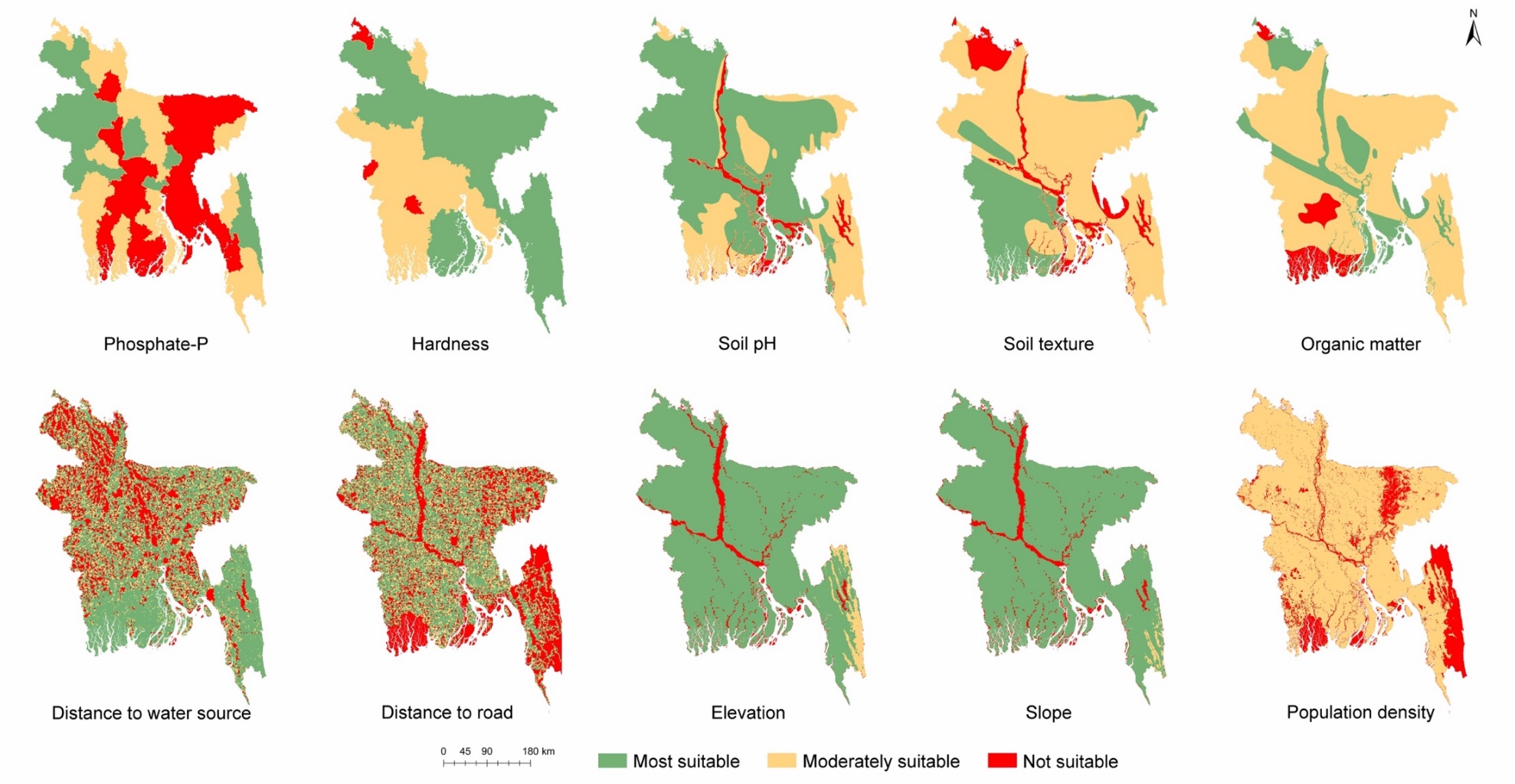


**Fig. S12.** Site suitability map for each dimension at 30m x 30m resolution.


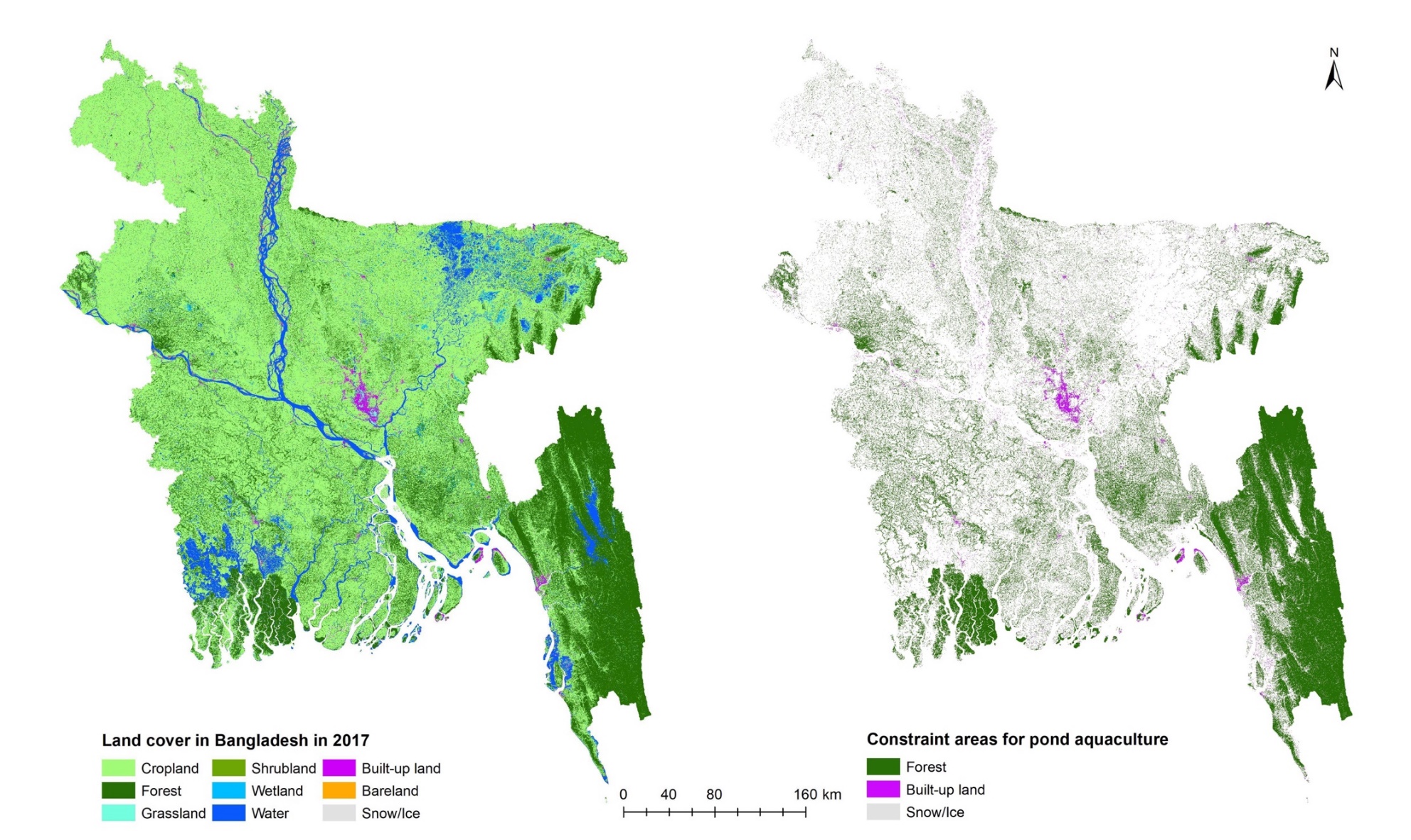


**Fig. S13.** Land cover map (Panel A) and forest and build-up areas (Panel B) at 10m x 10m resolution


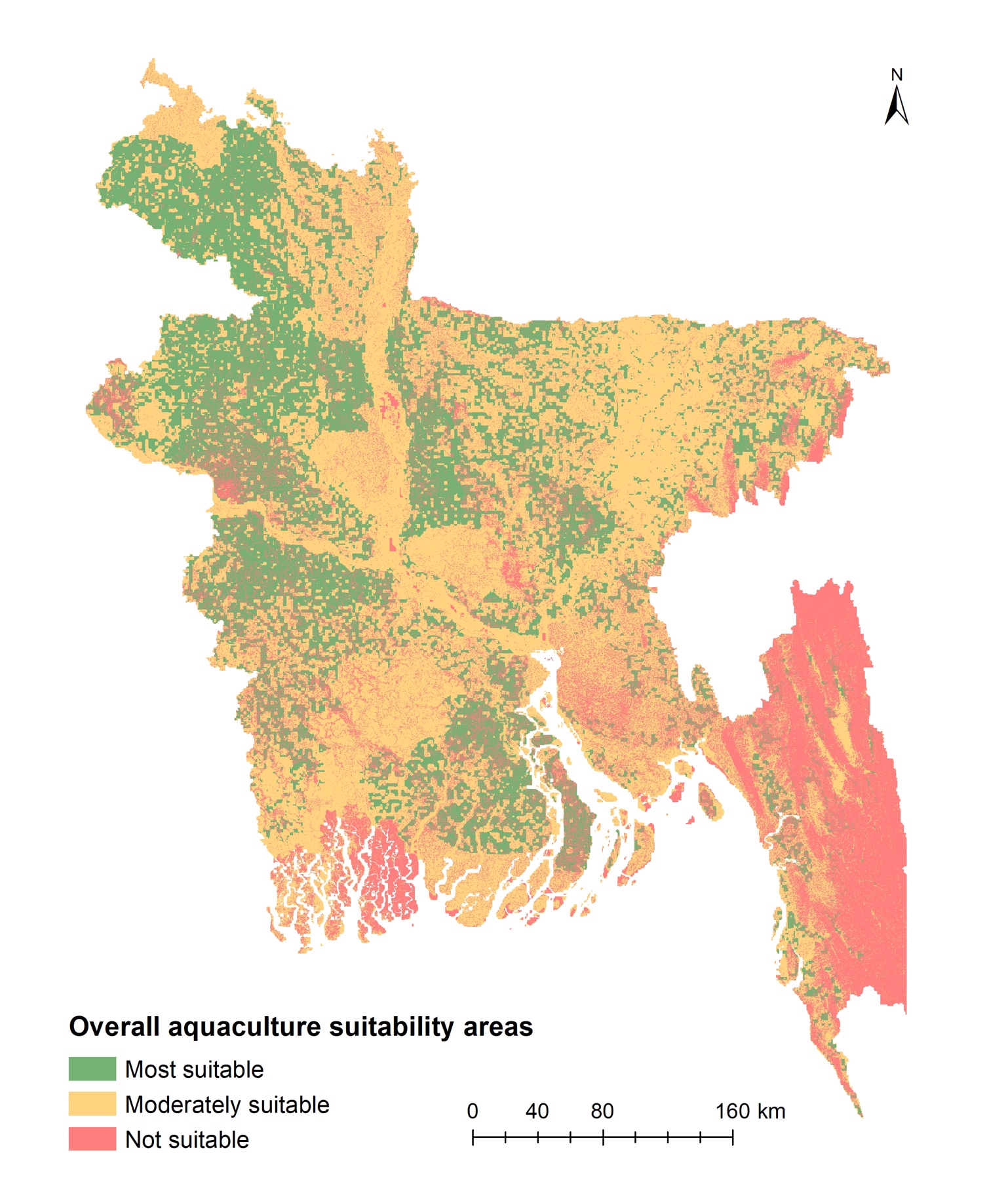


**Fig. S14**. Site suitability map for pond aquaculture in Bangladesh at 30m x 30m resolution


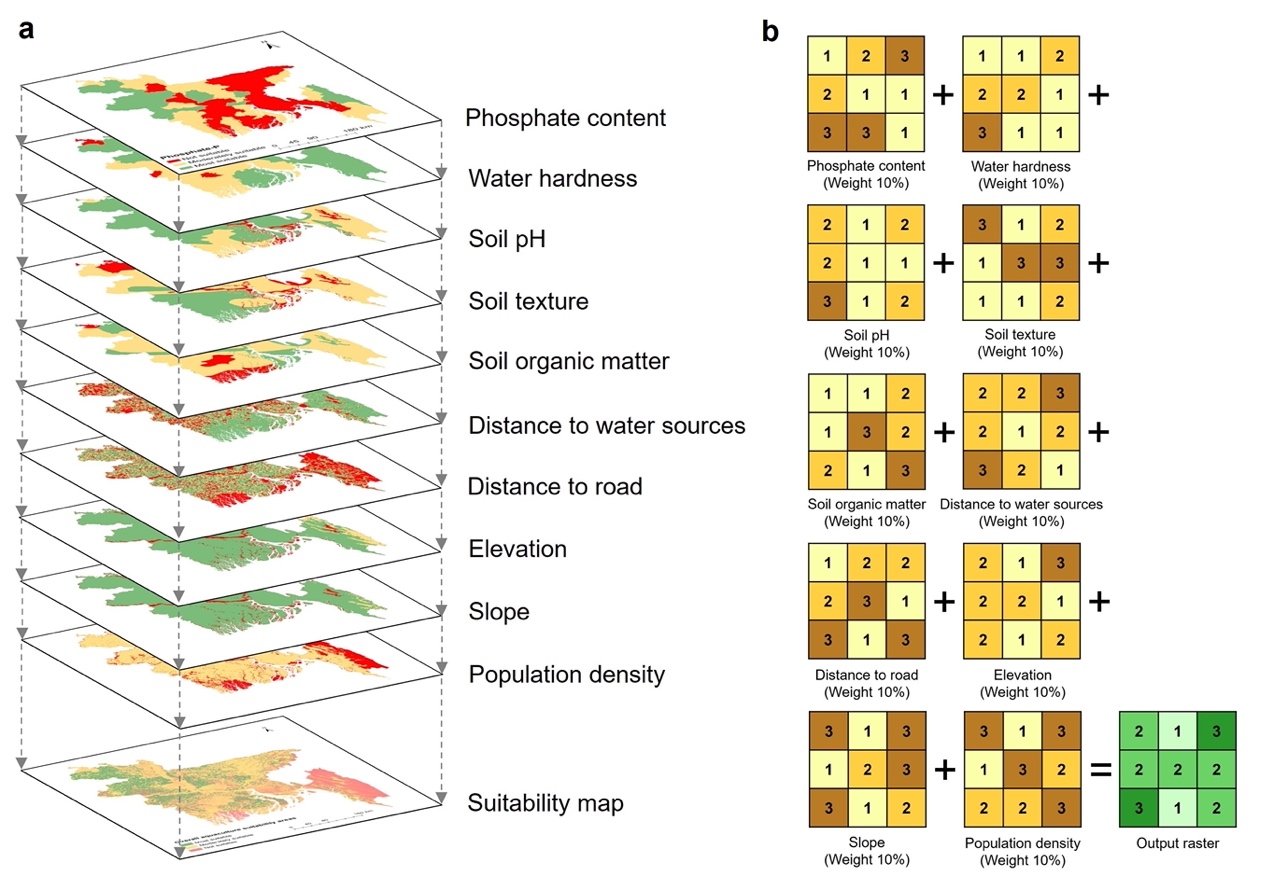


**Fig. S15.** Schematic representation of the weighted sum overlay analysis. Panel (a) shows the ten individual indicator layers following the resampling, application of cut-off suitability levels for each indicator and the re-classification. Panel (b) shows an example of the weighted sum overlay analysis. The yellow/brown matrices indicate suitability values for different cells. The green matrix indicates the output raster after the weighed summations for each pixel. Averages were rounded up or down to the closest whole number. Average values of <1.4 were rounded down to “1=no suitability”, average values of 1.5 to 2.4 were rounded up or down to “2=moderate suitability”; average values of >2.5 were rounded up to “3=high suitability”.


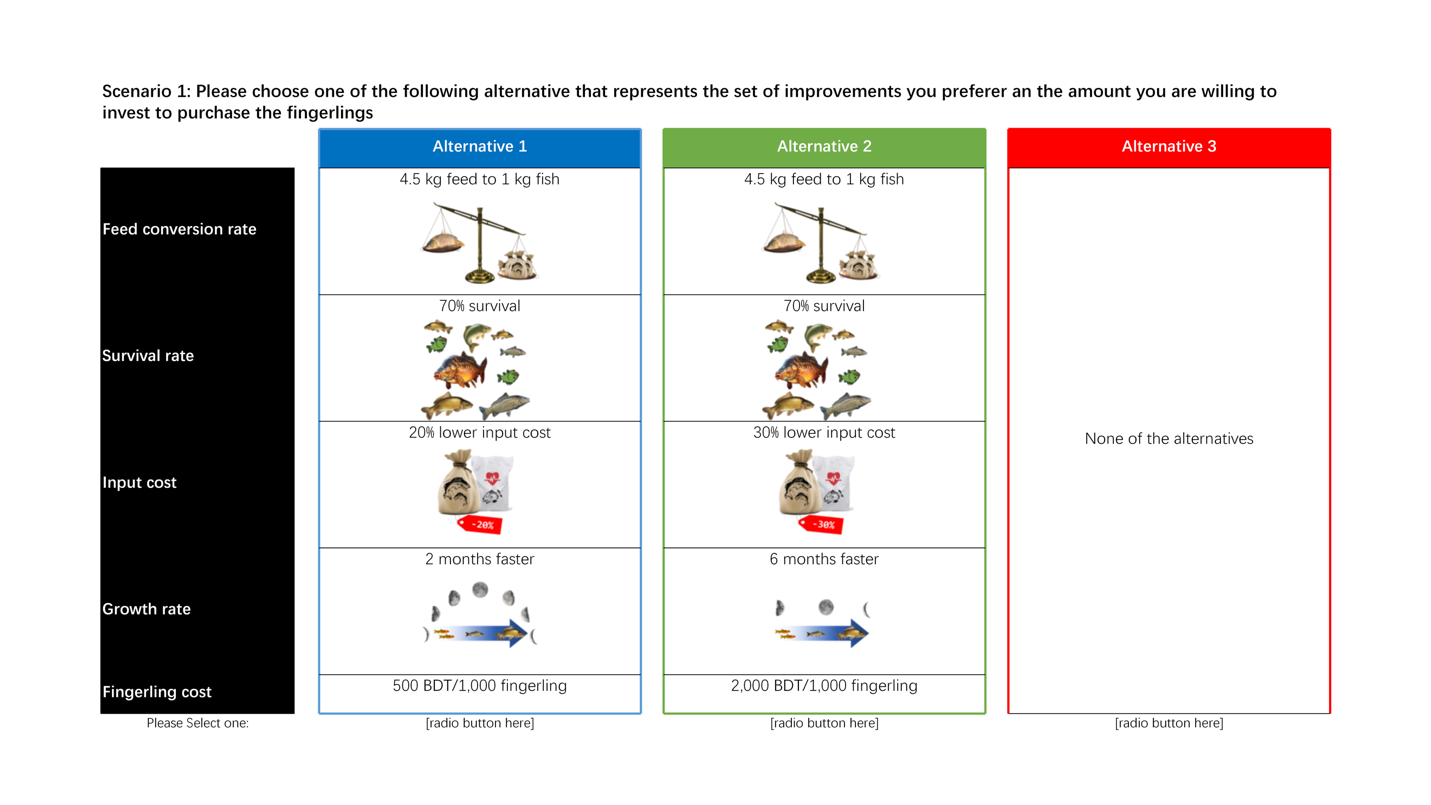


**Fig. S16.** Sample choice card for DCE1


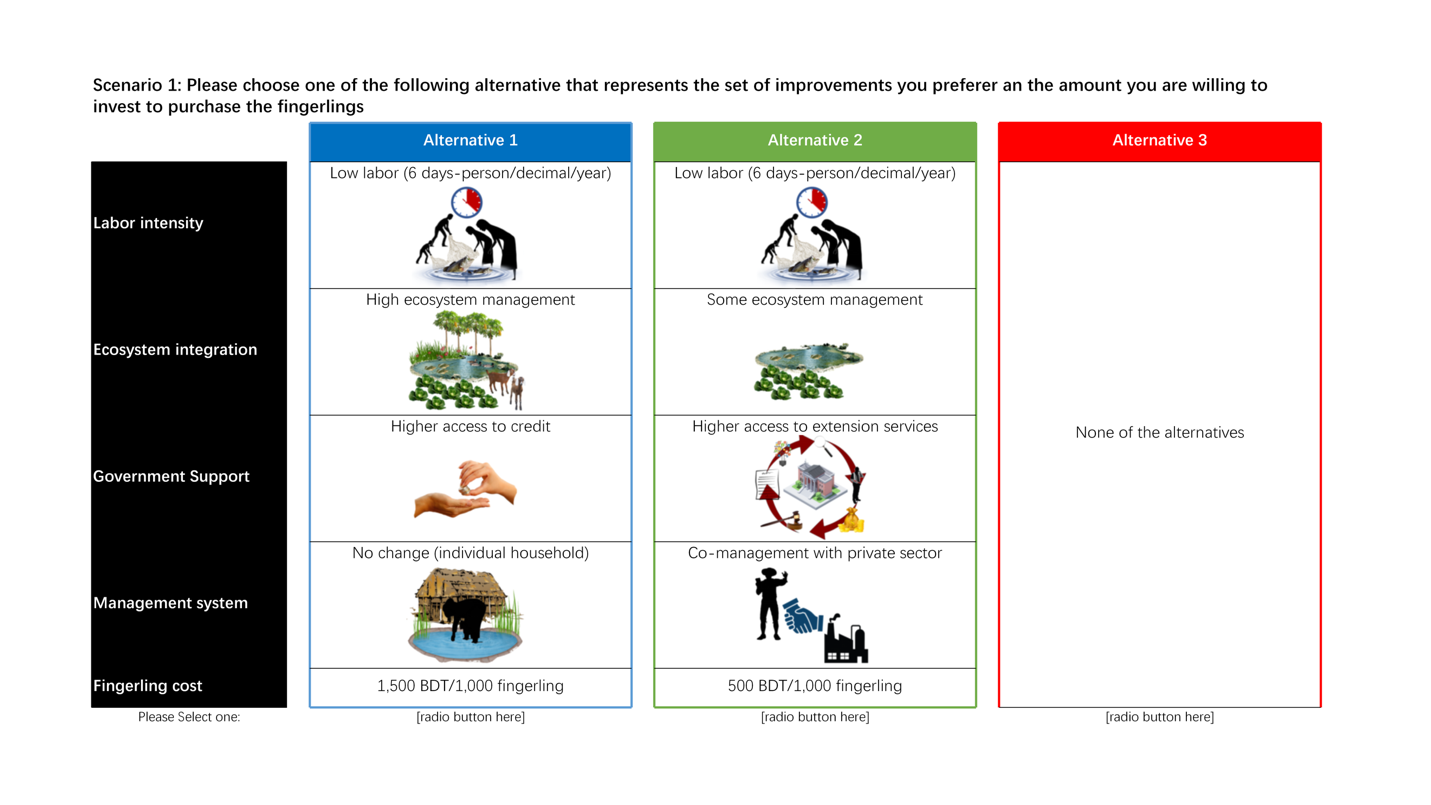


**Fig. S17.** Sample choice card for DCE2


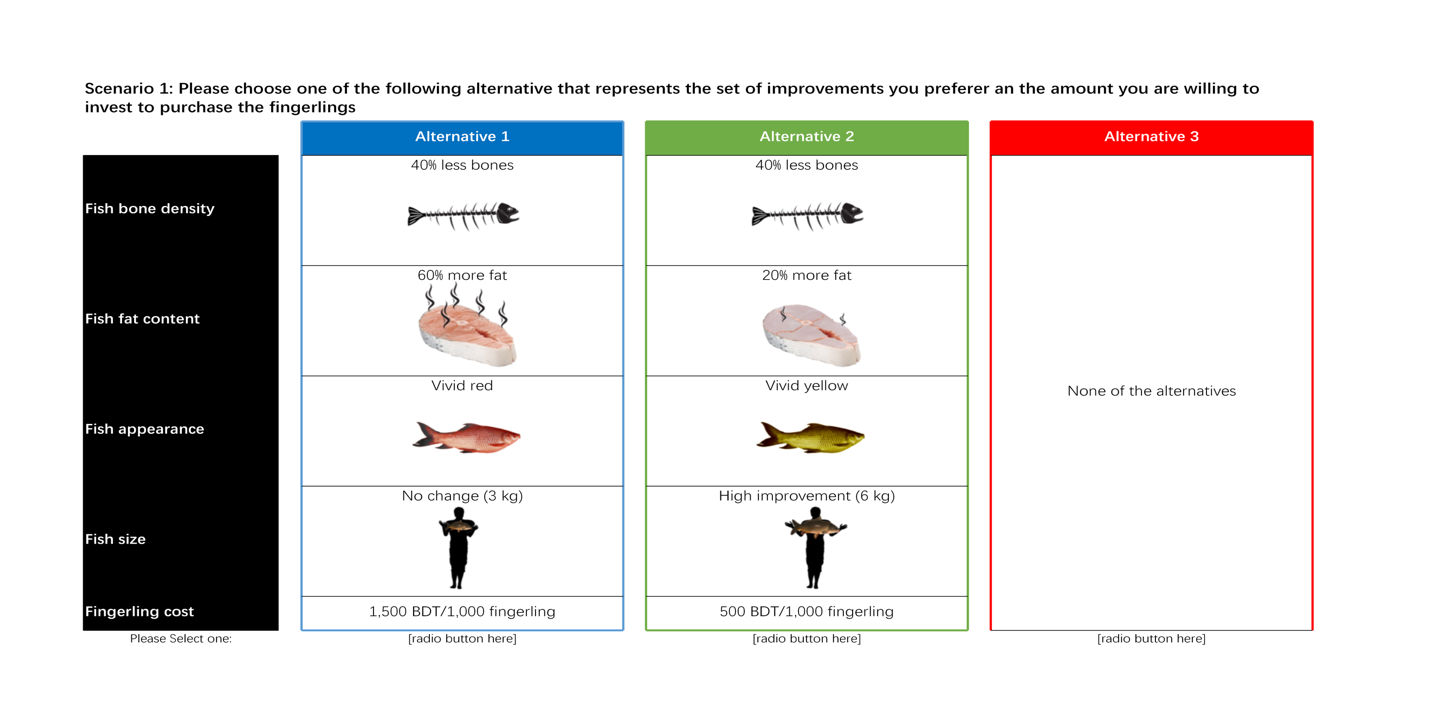


**Fig. S18.** Sample choice card for DCE3

**Table S1.** Indicators and suitability levels for aquaculture suitability analysis in Bangladesh.

| Suitability dimensions and indicators | Unit | Cut-off levels | | | Sources of cut-off levels | Data source |
| --- | --- | --- | --- | --- | --- | --- |
|  |  | High suitability | Moderate suitability | No suitability |  |  |
| ***Water quality*** | | | | | | |
| Phosphate content | mg/l | < 0.25 | 0.25-0.50 | > 0.50 | Nayak et al., 2018 | Bangladesh National Drinking Water Quality Survey(Johnston, 2011) |
| Water hardness | mg/l | 50-200 | 20-50 or  200-500 | < 20 or  > 500 | Nayak et al., 2018 | Bangladesh National Drinking Water Quality Survey(Johnston, 2011) |
| ***Soil quality*** | | | | | | |
| Soil pH |  | 6-8 | 4-6 or 8-9 | < 4 or > 9 | Hossain et al., 2009 | Harmonized World Soil Database(WIEDER, 2014) |
| Soil texture | % clay | > 35 | 18-35 | < 18 | Nayak et al., 2018 | Harmonized World Soil Database(WIEDER, 2014) |
| Soil organic matter | % of carbon | < 1 | 1-2 | > 2 | Hossain et al., 2009 | Harmonized World Soil Database(WIEDER, 2014) |
| ***Infrastructure*** | | | | | | |
| Distance to water source | m | < 500 | 500-1000 | > 1000 | Falconer et al., 2016  Nayak et al., 2018 | Humanitarian Data Exchange(“Bangladesh - Rivers,” 2018) |
| Distance to road | m | < 500 | 500-1000 | > 1000 | Hossain et al., 2009  Jayanthi et al., 2020 | Humanitarian Data Exchange(“Bangladesh - Roads,” 2018) |
| Elevation | m | < 150 | 150-1200 | > 2000 | Ghobadi et al., 2021 | Humanitarian Data Exchange(“Bangladesh - Contour Lines,” 2018) |
| Slope | % | < 5 | 5-15 | > 15 | Ghobadi et al., 2021 | Created by the elevation |
| Population density | Inhabitants per km^2^ | 150-300 | 1-149 | 1< or >300 | Berg et al., 2021 | WorldPop project(“Bangladesh - Population density,” 2015) |
| ***Constraints*** | | | | | | |
| Land cover | Land class |  |  | Built-up land, forest | Nayak et al., 2018  Jayanthi et al., 2020 | Global land cover in 2017(Gong et al., 2019) |

Note: Suitability levels for water quality, soil quality, and geographical characteristics were sourced from previous studies (Berg et al., 2021; Falconer et al., 2016; Ghobadi et al., 2021; Hossain et al., 2009; Jayanthi et al., 2020; Nayak et al., 2018). Datasets for each indicator were obtained from multiple sources (“Bangladesh - Contour Lines,” 2018; “Bangladesh - Population density,” 2015; “Bangladesh - Rivers,” 2018; “Bangladesh - Roads,” 2018; Johnston, 2011; WIEDER, 2014). Soil pH, soil texture and organic matter were collected at the topsoil (0-30 cm). Roads of Bangladesh include regional highways, upazila roads and union roads.

**Table S2** Areas (ha) and different suitability levels (%) of pond aquaculture in Bangladesh.

| Criteria | High suitability | | Moderate suitability | | Not suitable | |
| --- | --- | --- | --- | --- | --- | --- |
|  | ha | % | ha | % | ha | % |
| **Overall suitability** | 3609167.49 | 26.07 | 6672625.47 | 48.19 | 3564805.05 | 25.74 |

**Table S3**. Sample of respondents from Upazillas

| Upazila | Number of people in database | Interviewed respondents |
| --- | --- | --- |
| Adamdighi | 156 | 84 |
| Bagerhat Sadar | 150 | 84 |
| Bakerganj | 150 | 84 |
| Baraigram | 150 | 84 |
| Bauphal | 150 | 84 |
| Birampur | 153 | 84 |
| Birganj | 150 | 84 |
| Bochaganj | 152 | 84 |
| Chauddagram | 150 | 84 |
| Chaugachha | 152 | 84 |
| Chhatak | 135 | 84 |
| Daulatpur (Kushtia district) | 154 | 84 |
| Daulatpur (Manikganj) | 158 | 84 |
| Debidwar | 150 | 84 |
| Dewanganj | 150 | 84 |
| Gaffargaon | 150 | 86 |
| Gazipur Sadar | 150 | 85 |
| Gomastapur | 154 | 84 |
| Habiganj Sadar | 150 | 84 |
| Homna | 151 | 84 |
| Jagannathpur | 150 | 84 |
| Jaldhaka | 150 | 84 |
| Jhikargachha | 150 | 84 |
| Kahaloo | 150 | 84 |
| Kaharole | 153 | 85 |
| Kalmakanda | 152 | 84 |
| Kamalganj | 151 | 84 |
| Kamalnagar | 152 | 84 |
| Katiadi | 150 | 84 |
| Lakshmipur Sadar | 153 | 84 |
| Lalmohan | 150 | 83 |
| Magura Sadar | 150 | 84 |
| Manohardi | 150 | 84 |
| Manoharganj | 150 | 84 |
| Maulvi Bazar Sadar | 154 | 84 |
| Melandaha | 152 | 84 |
| Mirpur | 150 | 84 |
| Mitha Pukur | 150 | 84 |
| Mohanpur | 150 | 84 |
| Muradnagar | 156 | 84 |
| Mymensingh Sadar | 150 | 84 |
| Nilphamari Sadar | 150 | 83 |
| Pakundia | 131 | 84 |
| Patuakhali Sadar | 150 | 84 |
| Phulpur | 150 | 84 |
| Rajnagar | 151 | 86 |
| Roypura | 132 | 84 |
| Shibalaya | 150 | 84 |
| Shibganj | 151 | 84 |
| Singra | 150 | 84 |
| Sylhet Sadar | 117 | 84 |
| Tala | 151 | 84 |
| Trishal | 147 | 83 |
| Wazirpur | 148 | 85 |
| Total |  | 4540 |

**Table S4.** Production variables used for clustering.

| Variables | Form used | Unit | Description |
| --- | --- | --- | --- |
| Production area | Number | ha | Total size of all ponds used for aquaculture production in the previous production season. |
| Yield | Number | kg/ha | Fish output per pond unit area in the previous production season. This includes all fish species from all operational ponds (if more than one). |
| Feed frequency | Number | Times/day | Average number of times of fish feeding per day per day in the previous production season. This includes all fish species from all operational ponds (if more than one). |
| Distance to market | Number | km | Distance from home to the nearest market |
| Road condition | Dummy | Dummy  (good condition=1, otherwise=2) | Condition of the main road from the home to the nearest major town |
| Stocking density | Natural log | Number/ha | Number of fingerlings stocked per unit pond unit area in the previous production season. This includes all fish species from all operational ponds (if more than one). |
| Money borrowed for aquaculture | Natural log | USD | Amount of money borrowed for aquaculture from all sources in the previous production season. |
| Production cycle | Number | Number | Number of months of aquaculture production in the previous production season. |
| Family labour | Number | % | Family labour invested for all aquaculture activities in the previous production season, as a fraction of total farm labour for all aquaculture activities in the previous production season. This is for all operational ponds and aquaculture activities. |
| Adoption of Better Management Practices | Number | Average score | Average score of BMPs adopted for all ponds. |
| Carp dominated production | Dummy | Dummy  (Carp dominated=1, 0=otherwise) | Whether aquaculture production output in terms of mass is dominated by carp species. This includes all fish species from all operational ponds (if more than one).  Mass of carp species divided by total mass of aquaculture output from all operational ponds. If the fraction is >50% the household is described as carp-dominated. |
| Aquaculture asset cost | Natural log | USD | Current total cost of all assets used for aquaculture production. This is for all operational ponds and aquaculture activities. Assets might have been acquired before the previous production season, in contrast to expenditures (see below). |
| Weight of fingerlings | Natural log | Number | Average weight of fingerlings used for aquaculture production in the previous production season. This includes all fish species from all operational ponds (if more than one). |
| Commercial feed use | Number | kg/ha | Quantity of commercial mixed feed used per pond unit area in the previous production season. This includes all fish species from all operational ponds (if more than one). |
| Cooperative membership | Natural log | Number | Months of membership in aquaculture cooperatives. This does not include membership in other types of agriculture-, food- or livelihood-related cooperatives. |
| Commercialization index | Index | Number | Composite measure consisting of three equally weighed dimensions:  (a) % of fish output sold;  (b) % of total household income from aquaculture;  (c) % of aquaculture feed use that is commercial mixed feed  The estimates were for the previous production season. They covered all fish species from all operational ponds (if more than one). For income, all major and minor farm and off-farm income categories were included. |
| Aquaculture expenditure | Natural log | Number | Total amount of expenditures for all aquaculture in the previous production cycle. The estimates are for all aquaculture activities from all operational ponds (if more than one). |
| Labour cost | Natural log | USD/ha | Cost of hired labour per unit area in the previous production cycle. This included both permanent and seasonal labour for all aquaculture activities in all operational ponds (if more than one). |

**Table S5.** Production variables omitted from clustering due to collinearity.

| Variables | Form used | Unit | Description |
| --- | --- | --- | --- |
| Number of ponds | Number | Number | Number of ponds used for aquaculture production in the previous production season. This includes only operational ponds, and does not consider empty ponds (whether for aquaculture or other activities). |
| Weight at harvest | Number | gram/fish | Average weight of fish harvested in the previous production season. This includes all fish species from all operational ponds (if more than one). |
| Carp-generated value | Number | % | Value of sold carp output to the total value of all sold fish in the previous production season, estimated as a fraction. This considers only the value of fish sold, and not of the fish self-consumed. This includes all fish species from all operational ponds (if more than one). |
| Fish species diversity | Number | Number | Number of fish species produced in the previous production cycle. This is for all operational ponds (if more than one). |
| Tilapia-dominated aquaculture | Dummy | Dummy  (Tilapia dominated=1 0=otherwise) | Whether aquaculture production output in terms of mass is dominated by tilapia species. This includes all fish species from all operational ponds (if more than one).  Mass of carp species divided by total mass of aquaculture output from all operational ponds. If the fraction is >50% the household is described as carp-dominated. |
| Catfish dominated aquaculture (yes=1) | Dummy | Dummy  (Catfish dominated=1 0=otherwise) | Whether aquaculture production output in terms of mass is dominated by catfish species. This includes all fish species from all operational ponds (if more than one).  Mass of carp species divided by total mass of aquaculture output from all operational ponds. If the fraction is >50% the household is described as carp-dominated. |
| Permanent hired labour (percent labour hours) | Number | % | Permanent hired labour invested for all aquaculture activities in the previous production season, as a fraction of total farm labour for all aquaculture activities in the previous production season. Total labour includes permanent hired labour, seasonal hired labour, and family labour. This is for all operational ponds (if more than one). |

**Table S6.** Correlation matrix for variables used in the analysis

|  | Production area | Yieldkgha | Feed frequency | Commercial mixed feed | DISTANCE TOmarket | STATE OFROAD | Logstocking density | Average culture period | LOGweight  offingerlings | Logtotal  costofasset | Logaquacul  tureexpenditure | Carp dominated | AVERAGE BMP adoption | logmonthsofmembership | CommercializationIndex | Familylabour | logTotalcostoflabourperha | log_Moneyborrowed_aqua |
| --- | --- | --- | --- | --- | --- | --- | --- | --- | --- | --- | --- | --- | --- | --- | --- | --- | --- | --- |
| Production area | 1.0000 |  |  |  |  |  |  |  |  |  |  |  |  |  |  |  |  |  |
| Yieldkgha | -0.0503 | 1.0000 |  |  |  |  |  |  |  |  |  |  |  |  |  |  |  |  |
| Feedfrequency | 0.0123 | 0.0013 | 1.0000 |  |  |  |  |  |  |  |  |  |  |  |  |  |  |  |
| Commercial mixed feed | -0.0053 | 0.2430 | 0.0285 | 1.0000 |  |  |  |  |  |  |  |  |  |  |  |  |  |  |
| DISTANCETOmarket | 0.0940 | -0.0027 | -0.0030 | 0.0010 | 1.0000 |  |  |  |  |  |  |  |  |  |  |  |  |  |
| STATEOFROAD | 0.0014 | -0.0237 | 0.0007 | -0.0175 | 0.0465 | 1.0000 |  |  |  |  |  |  |  |  |  |  |  |  |
| Logstocking density | 0.1533 | 0.2173 | 0.0356 | 0.1595 | 0.0276 | -0.0141 | 1.0000 |  |  |  |  |  |  |  |  |  |  |  |
| Average culture period | 0.0214 | 0.0399 | -0.0023 | 0.0205 | 0.1028 | -0.0229 | 0.0001 | 1.0000 |  |  |  |  |  |  |  |  |  |  |
| LOGweightoffingerlings | 0.0261 | 0.0952 | -0.0039 | 0.0144 | 0.0693 | 0.0709 | -0.0025 | 0.0102 | 1.0000 |  |  |  |  |  |  |  |  |  |
| Logtotalcostofasset | 0.1687 | 0.1500 | 0.0244 | 0.1115 | 0.1100 | -0.0197 | 0.2560 | 0.0091 | 0.1296 | 1.0000 |  |  |  |  |  |  |  |  |
| Logaquacultureexpenditure | 0.1826 | 0.3571 | 0.0549 | 0.3434 | 0.0975 | -0.0193 | 0.4531 | 0.0398 | 0.1850 | 0.5135 | 1.0000 |  |  |  |  |  |  |  |
| Carp dominated | -0.0171 | -0.1219 | -0.0083 | -0.1781 | 0.0282 | 0.0027 | -0.1385 | 0.0189 | 0.0671 | -0.0091 | -0.0562 | 1.0000 |  |  |  |  |  |  |
| AVERAGE BMP adoption | 0.0634 | 0.1341 | 0.0343 | 0.0645 | 0.0983 | -0.0703 | 0.1003 | 0.0040 | -0.0015 | 0.2083 | 0.2579 | 0.0867 | 1.0000 |  |  |  |  |  |
| logmonthsofmembership | -0.0073 | -0.0438 | 0.0249 | 0.0064 | 0.0521 | 0.0002 | 0.0012 | 0.0057 | -0.0391 | 0.0788 | 0.0292 | 0.0222 | 0.1092 | 1.0000 |  |  |  |  |
| CommercializationIndex | 0.1207 | 0.2901 | 0.0471 | 0.3655 | 0.0591 | -0.0442 | 0.3950 | 0.0150 | 0.0375 | 0.3271 | 0.6016 | -0.1326 | 0.2655 | 0.0637 | 1.0000 |  |  |  |
| Familylabour | -0.1560 | -0.0432 | 0.0077 | -0.0987 | -0.0992 | 0.0090 | -0.2239 | -0.0238 | -0.0849 | -0.2368 | -0.4005 | 0.0240 | -0.1548 | 0.0188 | -0.2091 | 1.0000 |  |  |
| logTotalcostoflabourperha | -0.0441 | 0.2601 | -0.0082 | 0.1358 | 0.0143 | -0.0333 | 0.1737 | 0.0236 | 0.1250 | 0.2064 | 0.4869 | -0.0506 | 0.2016 | 0.0099 | 0.2211 | -0.5225 | 1.0000 |  |
| log_Moneyborrowed_aqua | 0.0338 | 0.1186 | 0.0202 | 0.1353 | 0.0929 | -0.0273 | 0.2015 | 0.0168 | 0.1219 | 0.1499 | 0.3194 | -0.0572 | 0.1359 | 0.0044 | 0.2547 | -0.1456 | 0.1927 | 1.0000 |

**Table S7.** Principal component analysis estimation

| Component | Eigenvalue | Difference | Proportion | Cumulative |
| --- | --- | --- | --- | --- |
|  |  |  |  |  |
| Comp1 | 3.46849 | 2.07901 | 0.1927 | 0.1927 |
| Comp2 | 1.38949 | .170699 | 0.0772 | 0.2699 |
| Comp3 | 1.21879 | .0748413 | 0.0677 | 0.3376 |
| Comp4 | 1.14395 | .0444824 | 0.0636 | 0.4012 |
| Comp5 | 1.09946 | .0516018 | 0.0611 | 0.4622 |
| Comp6 | 1.04786 | .0587663 | 0.0582 | 0.5204 |
| Comp7 | .989097 | .0105048 | 0.0549 | 0.5754 |
| Comp8 | .978592 | .0666112 | 0.0544 | 0.6298 |
| Comp9 | .911981 | .0390883 | 0.0507 | 0.6804 |
| Comp10 | .872893 | .03927 | 0.0485 | 0.7289 |
| Comp11 | .833623 | .0399233 | 0.0463 | 0.7752 |
| Comp12 | .793699 | .0388327 | 0.0441 | 0.8193 |
| Comp13 | .754867 | .0561975 | 0.0419 | 0.8613 |
| Comp14 | .698669 | .0637041 | 0.0388 | 0.9001 |
| Comp15 | .634965 | .136391 | 0.0353 | 0.9354 |
| Comp16 | .498574 | .0902 | 0.0277 | 0.9631 |
| Comp17 | .408374 | .151753 | 0.0227 | 0.9857 |
| Comp18 | .256621 | . | 0.0143 | 1.0000 |
|  |  |  |  |  |

Note: Principal components/correlation: Number of obs = 4,211; Number of comp. = 18; Trace = 18; Rotation: (unrotated = principal) Rho = 1.0000

**Table S8.** Principal component of eigen values

|  | |  | |  | |  | |  | |  | |  |  |  |  |  |  |  |  |  |  |  |  |  |  |
| --- | --- | --- | --- | --- | --- | --- | --- | --- | --- | --- | --- | --- | --- | --- | --- | --- | --- | --- | --- | --- | --- | --- | --- | --- | --- |
| Variable | Comp1 | | Comp2 | | Comp3 | | Comp4 | | Comp5 | | Comp6 | | Comp7 | Comp8 | Comp9 | Comp10 | Comp11 | Comp12 | Comp13 | Comp14 | Comp15 | Comp16 | Comp17 | Comp18 | Unexplained |
| Production~a | 0.1146 | | 0.2500 | | 0.3966 | | 0.4353 | | -0.3124 | | -0.0715 | | 0.1528 | -0.0901 | 0.1204 | -0.0831 | -0.1115 | 0.1933 | 0.4126 | 0.3358 | 0.2481 | 0.0046 | 0.1337 | 0.0988 | 0 |
| Yieldkgha | 0.2490 | | -0.3169 | | -0.1825 | | -0.0947 | | 0.2784 | | -0.0155 | | 0.1068 | -0.1286 | 0.2598 | 0.1177 | -0.1700 | -0.3908 | 0.1553 | 0.4008 | 0.3827 | 0.0641 | -0.2999 | 0.0364 | 0 |
| Feedfreque~y | 0.0318 | | -0.0373 | | 0.2182 | | -0.0574 | | 0.0843 | | 0.3260 | | 0.3585 | 0.8303 | -0.0156 | 0.0104 | -0.0940 | -0.0523 | -0.0101 | 0.0289 | 0.0404 | 0.0058 | -0.0146 | 0.0256 | 0 |
| Commerc~a_kg | 0.2350 | | -0.4047 | | 0.0432 | | 0.0070 | | 0.1959 | | -0.0431 | | -0.0876 | 0.0090 | 0.0358 | 0.0141 | -0.2191 | 0.6949 | -0.1435 | 0.0237 | -0.0069 | 0.4079 | 0.0460 | 0.0951 | 0 |
| DISTANCETO~T | 0.0832 | | 0.3520 | | 0.1919 | | 0.1862 | | 0.3644 | | -0.2672 | | -0.1797 | 0.0438 | -0.3412 | 0.3187 | -0.4318 | -0.1663 | -0.3183 | 0.1366 | -0.0143 | -0.0279 | 0.0774 | 0.0184 | 0 |
| STATEOFROAD | -0.0285 | | 0.0779 | | -0.0997 | | 0.4338 | | 0.2573 | | 0.3805 | | -0.5021 | 0.1391 | 0.2931 | 0.3430 | 0.2756 | 0.0414 | 0.1485 | -0.0827 | 0.0469 | -0.0039 | 0.0044 | 0.0160 | 0 |
| logstockin~y | 0.3063 | | -0.1434 | | 0.1881 | | 0.1898 | | -0.1824 | | 0.0138 | | 0.0336 | -0.0330 | 0.0408 | 0.0273 | 0.3435 | -0.3561 | -0.2681 | 0.2516 | -0.4722 | 0.3841 | 0.0954 | 0.1286 | 0 |
| Average_cu~s | 0.0293 | | 0.1067 | | 0.0339 | | 0.0444 | | 0.3774 | | -0.6956 | | 0.0861 | 0.2507 | 0.3319 | -0.1817 | 0.3208 | 0.0204 | 0.1316 | -0.1411 | -0.0641 | 0.0086 | -0.0048 | 0.0118 | 0 |
| LOG_FINGER~g | 0.1055 | | 0.2342 | | -0.3647 | | 0.2556 | | 0.3721 | | 0.2754 | | 0.2433 | -0.1309 | -0.0721 | -0.4427 | -0.2032 | 0.0246 | 0.1837 | 0.0481 | -0.4000 | 0.0265 | -0.0356 | 0.0622 | 0 |
| logtotalco~t | 0.3111 | | 0.1989 | | 0.1649 | | 0.0484 | | -0.0201 | | 0.1422 | | 0.0604 | -0.1480 | 0.1780 | -0.1870 | -0.1023 | -0.1897 | -0.2173 | -0.6331 | 0.3410 | 0.2176 | -0.0703 | 0.2413 | 0 |
| LOGAQUAEXP~s | 0.4684 | | 0.0216 | | -0.0131 | | 0.0339 | | 0.0055 | | 0.0605 | | 0.0489 | -0.0218 | 0.0976 | -0.0471 | 0.0284 | 0.0330 | -0.1144 | -0.0315 | 0.0611 | -0.1567 | 0.2436 | -0.8105 | 0 |
| Carp_domin~d | -0.0828 | | 0.4719 | | -0.0601 | | -0.2602 | | 0.1197 | | 0.1675 | | 0.3134 | -0.1634 | 0.2793 | 0.1834 | 0.2705 | 0.2808 | -0.3952 | 0.3047 | 0.1102 | 0.0218 | -0.0123 | 0.0796 | 0 |
| AVERAGEBMP~E | 0.2114 | | 0.2177 | | 0.1724 | | -0.4566 | | 0.0517 | | 0.0506 | | 0.0399 | -0.1039 | 0.0286 | 0.4192 | -0.0850 | -0.0014 | 0.5342 | -0.1783 | -0.3191 | 0.2176 | 0.0008 | -0.0429 | 0 |
| log_months~p | 0.0299 | | 0.1363 | | 0.3663 | | -0.3832 | | 0.1726 | | 0.1629 | | -0.5295 | 0.0297 | -0.0146 | -0.5264 | 0.0694 | -0.0344 | 0.0296 | 0.2689 | 0.0461 | 0.0270 | -0.0475 | -0.0211 | 0 |
| Commercial~I | 0.3840 | | -0.1800 | | 0.2218 | | -0.0280 | | 0.0649 | | 0.0480 | | 0.0251 | -0.1001 | 0.0458 | 0.0699 | 0.0599 | 0.1397 | -0.0671 | -0.0426 | -0.2121 | -0.7390 | -0.1366 | 0.3268 | 0 |
| Familylabour | -0.2891 | | -0.2837 | | 0.2792 | | -0.0269 | | 0.3893 | | 0.1439 | | 0.2063 | -0.2556 | 0.0242 | 0.0026 | 0.0527 | -0.1474 | 0.0553 | -0.0647 | 0.0781 | -0.0520 | 0.6576 | 0.0593 | 0 |
| logTota~aUSD | 0.3165 | | 0.1158 | | -0.4690 | | -0.2067 | | -0.1780 | | -0.0768 | | -0.1845 | 0.2143 | 0.0105 | -0.0234 | -0.0426 | -0.0421 | 0.0521 | 0.0966 | 0.1218 | -0.0657 | 0.5908 | 0.3489 | 0 |
| log_Moneyb~a | 0.2350 | | 0.0129 | | -0.0409 | | 0.0352 | | 0.1777 | | 0.0221 | | 0.1163 | -0.0375 | -0.6886 | 0.0312 | 0.5221 | 0.0835 | 0.1599 | -0.0118 | 0.3143 | 0.0818 | -0.0811 | 0.0475 | 0 |

**Table S9.** Kaiser-Meyer-Olkin measure of sampling adequacy

| **Variable** | **kmo** |
| --- | --- |
|  |  |
| Production area | 0.6055 |
| Yieldkgha | 0.7916 |
| Feedfrequency | 0.6751 |
| Commercial mixed feed | 0.7921 |
| Distance to market | 0.6646 |
| State of road | 0.5584 |
| Logstocking density | 0.8460 |
| Average culture period | 0.5405 |
| LOGweightoffingerlings | 0.6618 |
| Logtotalcostofasset | 0.8185 |
| Logaquacultureexpenditure | 0.7664 |
| Carp dominated | 0.5931 |
| AVERAGE BMP adoption | 0.7962 |
| logmonthsofmembership | 0.5279 |
| CommercializationIndex | 0.8184 |
| Familylabour | 0.7137 |
| logTotalcostoflabourperha | 0.6732 |
| log_Moneyborrowed_aqua | 0.8891 |
|  |  |
| Overall | 0.7642 |

**Table S10.** Optimum number of clusters determination (Calinski/Harabasz)

| Number of clusters | Calinski/ Harabasz pseudo-F |
| --- | --- |
| 2 | 993.15 |
| 3 | 708.18 |
| 4 | 614.87 |
| 5 | 546.80 |
| 6 | 490.26 |
| 7 | 455.90 |
| 8 | 527.50 |
| 9 | 496.68 |
| 10 | 476.97 |
| 11 | 455.57 |
| 12 | 455.68 |
| 13 | 436.89 |
| 14 | 472.37 |
| 15 | 453.01 |

**Table S11.** Optimum number of clusters determination (Duda/Hart rule)

| Number of clusters | Duda/Hart | |
| --- | --- | --- |
|  | Je(2)/Je(1) | pseudo T-squared |
| 1 | 0.8091 | 993.15 |
| 2 | 0.8712 | 382.77 |
| 3 | 0.8424 | 302.78 |
| 4 | 0.8996 | 235.23 |
| 5 | 0.7419 | 336.81 |
| 6 | 0.8887 | 142.47 |
| 7 | 0.4560 | 281.54 |
| 8 | 0.7449 | 308.26 |
| 9 | 0.9138 | 122.85 |
| 10 | 0.8984 | 100.30 |
| 11 | 0.7831 | 120.50 |
| 12 | 0.8052 | 162.32 |
| 13 | 0.4364 | 173.08 |
| 14 | 0.8062 | 114.91 |
| 15 | 0.8468 | 74.89 |

**Table S12.** Methodological approach for outcome variables.

| Variable | Formula | Description |
| --- | --- | --- |
| *Economic performance* |  |  |
| Fish sales per unit area | $Y=\frac{\sum_{i,j=1}^{n} F_{i,j}}{A}$ | $F_{i,j}$ indicates the total amount of fish income regarding fish species $i$ in pond $j$; $A$ denotes the total pond area for each household. |
| Aquaculture benefit-cost ratio (BCR) | $BCR=\frac{B}{C}$ | $BCR$ indicates benefit-cost ratio of aquaculture production. $B$ is the total economic benefits from aquaculture production. $C$ indicates the total aquaculture costs, including labor, equipment, pond rentals, material input, freshwater and electricity consumption, transportation. |
| Income per capita | $Y=\frac{\sum_{i=1}^{n} I_{i}}{M}$ | $I_{i}$ denotes the total amount of income $i$ in each household; $M$ denotes total number of household members. |
| *Social performance* |  |  |
| Fish self-consumption per capita | $FC=\frac{\sum_{i=1}^{n} F_{i}}{M}$ | $F_{i}$ denotes the quantities of household consumption regarding fish species $i$, $i$ = 1, 2, …, $n$; $M$ denotes total number of household members. |
| Food consumption score (FCS) | $FCS=\sum_{i=1}^{n} F_{i}\times W_{i}$ | $F_{i}$ denotes food group $i$, $i$ = 1, 2, …, $n$. $W_{i}$ denotes the weight of food group $i$ (WFP, 2008). Food group weights are included in Table S14. |
| Deprivation scores | $DS=\sum_{a=1}^{n} \frac{1}{6}\times E_{a}+\sum_{b=1}^{n} \frac{1}{6}\times H_{b}+\sum_{c=1}^{n} \frac{1}{18}\times L_{c}$ | $E_{a}$ indicates education variable $a$ in deprivation dimension of education; $H_{b}$ indicates health variable $b$ in deprivation dimension of health; $L_{c}$ indicates living standard variable $c$ in deprivation dimension of living standards. Dimensions, indicators, cut-off of deprivation, and weights are outlined in Table S15. |
| *Environmental performance* |  |  |
| Nitrogen and phosphorus use efficiency | $E_{N,P}=\frac{\sum_{k=1}^{m} F_{k}S_{k}}{\sum_{i,j=1}^{n} F_{i}R_{i}{+G}_{j}C_{j}}$ | $P_{k}$ indicates the total production of fish $k$ (kg), and $S_{k}$ denotes the nitrogen or phosphorus composition rate (%) of fish $k$. $F_{i}$ indicates the input from feed $i$ (kg) (e.g. rice bran, meat meal), $R_{i}$ denotes the nitrogen or phosphorus composition rate (%) of feed $i$. $G_{j}$ is the input from fertilizer $j$ (kg) (e.g. cow dung, poultry droppings, urea, compound fertilizer), $C_{i}$ is the nitrogen or phosphorus composition rate (%) of fertilizer $j$. The coefficients related to the nitrogen or phosphorus composition of feed, fertilizer, and fish species are shown in Table S15. |

**Table S13.** Food groups and weights in the calculation of food consumption score (FCS).

| Food group | Food item | Weight |
| --- | --- | --- |
| Main staples | e.g., rice, wheat, maize, and other items food made from these cereals | 2 |
| Pulses | e.g., bean, chickpea, groundnut | 3 |
| Vegetables | e.g., potato, tomato, brinjal, okra, bitter gourd, cauliflower, cabbages, and leafy vegetables | 1 |
| Fruits | e.g., mango, banana, watermelon, pineapple, guava | 1 |
| Meat/fish | e.g., beef, mutton, poultry, pork, eggs, fish | 4 |
| Milk and diary | e.g., cow milk, buffalo milk, goat milk, yogurt, cheese, milk powder | 4 |
| Sugar/honey | e.g., sugar and sugar products | 0.5 |
| Oil | e.g., oil, fat, butter | 0.5 |

Note: Data were sourced from WFP (WFP, 2008).

**Table S14.** The dimensions, indicators, weights and cut-offs of deprivation scores.

| Dimension | Indicator | Cut-off deprivation | Weight |
| --- | --- | --- | --- |
| Education | Years of schooling | Deprived if no household member has completed 5 years of schooling | 1/6 |
|  | Child school attendance | Deprived if any school-aged child is not attending school in years 1 to 8 | 1/6 |
| Health | Nutrition | Deprived if the food consumption score is below acceptable threshold (63 or below) | 1/6 |
|  | Child mortality | Deprived if any under-5year old child died in the household during past 12 months preceding census | 1/6 |
| Living standards | Electricity | Deprived if the household has no electricity. | 1/18 |
|  | Drinking water | Deprived if the household does not have access to safe drinking water or safe drinking water is more than 30 minutes round trip walk from homestead | 1/18 |
|  | Sanitation | Deprived if the household’s sanitation facility is not improved | 1/18 |
|  | Flooring | Deprived if a household has sand, dirt and or dung floor | 1/18 |
|  | Cooking fuel | Deprived if the household cooks with firewood, dung and charcoal | 1/18 |
|  | Assets ownership | Deprived if the household does not own more than one radio, TV, telephone, bike, motorbike or refrigerator, car or truck or tractor | 1/18 |

Note: Data were sourced from a recent study (Alkire and Santos, 2014). A household is deemed to have access to upgraded sanitation if it possesses a flush toilet, latrine, ventilated improved pit, or composting toilet, ensuring individual use without sharing. A household ensures safe drinking water access through piped water, public taps, boreholes, pumps, protected wells, springs, or rainwater sources.

**Table S15.** Nutrient compositions of fertilizers, feeds, and fish species in Bangladesh.

| Category | Material | Composition (%) | | Types of data sources | Citation |
| --- | --- | --- | --- | --- | --- |
|  |  | N | P |  |  |
| Farm by-product fertilizer | Cow dung | 0.75 | 0.15 | Local sources | Barman and Karim (2007) |
|  | Poultry droppings | 1.90 | 0.56 | Local sources | Barman and Karim (2007) |
|  | Compost | 0.75 | 0.60 | Local sources | Barman and Karim (2007) |
| Inorganic fertilizer | Urea | 46.70 | 0 | General sources | Tacon et al. (2009) |
|  | Triple super phosphate (TSP) | 0 | 19.21 | General sources | Donovan et al. (2000); Tacon et al. (2009) |
|  | Di-ammonium phosphate (DAP) | 21.20 | 23.50 | General sources | Tacon et al. (2009) |
|  | Compound fertilizer | 10.00 | 10.92 | General sources | Wiseman et al. (2014) |
| Farm by-product feed | Rice bran | 1.34 | 1.73 | Local and general sources | Agboola et al. (2019); Green (2015) |
|  | Groundnut cake | 5.14 | 1.17 | General sources | Tacon et al. (2009); Settaluri et al. (2012) |
|  | Wheat bran | 2.62 | 1.28 | Local and general sources | Agboola et al. (2019); Tacon et al. (2009) |
|  | Maize bran | 1.81 | 0.28 | Local and general sources | Tacon et al. (2009); Barman and Karim (2007) |
|  | Coconut meal | 3.01 | 0.60 | Local and general sources | Agboola et al. (2019); Tacon et al. (2009) |
|  | Soy cake | 6.83 | 0.62 | General sources | Tacon et al. (2009) |
|  | Snails | 8.62 | 0.49 | Local and general sources | Agboola et al. (2019); Tacon et al. (2009) |
|  | Molasses | 0.72 | 5.30 | Local and general sources | Tacon et al. (2009); Barman and Karim (2007) |
|  | Dahl | 3.90 | 0.31 | General sources | Tacon et al. (2009) |
|  | Corn gluten meal | 8.98 | 0.44 | General sources | Tacon et al. (2009) |
|  | Mustard oil cake | 5.20 | 1.14 | Local and general sources | Agboola et al. (2019); Tacon et al. (2009) |
|  | Azolla/Duckweed | 3.38 | 0.20 | Local and general sources | Agboola et al. (2019); Tacon et al. (2009) |
|  | Nursery/starter feed | 4.53 | 0.49 | Local and general sources | Ahme (2013); Rahman et al. (2014); Tacon et al. (2009) |
|  | Own farm-made pelleted feed | 4.46 | 0.80 | General sources | Tacon et al. (2009); Ahme (2013) |
|  | Own farm-made mixed feed | 3.15 | 1.21 | Local and general sources | Agboola et al. (2019); Tacon et al. (2009); Barman and Karim (2007) |
| Commercial feed | Commercial feed pellets (floating and sinking pellets) | 9.46 | 0.40 | General sources | Tacon et al. (2009); Saba and Steinberg (2012); M. Abdelhamid et al. (2019) |
|  | Fish meal | 9.02 | 2.82 | Local and general sources | Barman and Karim (2007); Tacon et al. (2009) |
|  | Shrimp feed | 7.73 | 2.02 | Local and general sources | Agboola et al. (2019); Tacon et al. (2009) |
| Fish species | Rui (*Labeo rohita*) | 2.91 | 0.21 | Local sources | Bogard et al. (2015) |
|  | Catla (*Catla catla*) | 3.18 | 0.24 | Local sources | Shaheen et al. (2013) |
|  | Mrigal (*Cirrhinus cirrhosus*) | 2.98 | 0.28 | Local sources |  |
|  | Common carp (*Cyprinus carpio*) | 2.99 | 0.24 | Local sources |  |
|  | Grass carp (*Ctenopharyngodon idella*) | 2.43 | 0.19 | Local sources | Bogard et al. (2015) |
|  | Silver carp (*Hypophthalmichthys molitrix*) | 2.80 | 0.18 | Local sources | Shaheen et al. (2013) |
|  | Tilapia (*Oreochromis*  *mossambicus*) | 3.33 | 0.35 | Local sources | Shaheen et al. (2013) |
|  | Sorpunti (*Puntius sarana*) | 2.78 | 0.15 | Local sources | Shaheen et al. (2013) |
|  | Bata (*Labeo bata*) | 2.54 | 0.20 | Local sources | Shaheen et al. (2013) |
|  | Koi (*Anabas testudineus*) | 2.80 | 0.39 | Local sources | Shaheen et al. (2013) |
|  | Kalbaush (*Labeo calbasu*) | 2.72 | 0.14 | Local sources | Shaheen et al. (2013) |
|  | Gonia (*Labeo gonius*) | 2.82 | 0.18 | Local sources | Shaheen et al. (2013) |
|  | Sliver barb (*Barbonymus gonionotus*) | 2.94 | 0.28 | Local sources | Shaheen et al. (2013) |
|  | Magur (*Clarias batrachus*) | 2.50 | 0.18 | Local sources | Shaheen et al. (2013) |
|  | Pangas (*Pangasius pangasius*) | 2.54 | 0.13 | Local sources | Shaheen et al. (2013) |
|  | Boal (*Wallago attu*) | 2.46 | 0.13 | Local sources | Shaheen et al. (2013) |
|  | Tengra (*Mystus tengara*) | 2.91 | 0.35 | Local sources | Shaheen et al. (2013) |
|  | Taki (*Channa punctatus*) | 2.77 | 0.44 | Local sources | Shaheen et al. (2013) |
|  | Shol (*Channa striata*) | 2.83 | 0.13 | Local sources | Shaheen et al. (2013) |
|  | Ayre (*Mystus aor*) | 2.72 | 0.10 | Local sources | Shaheen et al. (2013) |
|  | Mola (*Amblypharyngodon*  *mola*) | 2.74 | 0.44 | Local sources | Shaheen et al. (2013) |
|  | Punti (*Puntius sophore*) | 2.82 | 0.62 | Local sources | Shaheen et al. (2013) |
|  | Chela (*Salmophasia phulo*) | 2.45 | 0.22 | Local sources | Shaheen et al. (2013) |
|  | Khailsa (*Colisa fasciata*) | 2.53 | 0.50 | Local sources | Shaheen et al. (2013) |
|  | Pabda (*Ompok pabda*) | 2.77 | 0.27 | Local sources | Shaheen et al. (2013) |
|  | Chingri (*Macrobrachium rude*) | 2.82 | 0.14 | Local sources | Shaheen et al. (2013) |
|  | Chingri, Bagda (*Penaeus monodon*) | 2.64 | 0.14 | Local sources | Shaheen et al. (2013) |
|  | Chingri, Golda (*Macrobrachium*  *rosenbergii*) | 3.34 | 0.17 | Local sources | Shaheen et al. (2013) |

Note: Data were obtained from multiple sources (Agboola et al., 2019; Ahme, 2013, 2013; Barman and Karim, 2007; Bogard et al., 2015; Donovan et al., 2000; Green, 2015; M. Abdelhamid et al., 2019; Rahman et al., 2014; Saba and Steinberg, 2012; Settaluri et al., 2012; Shaheen et al., 2013; Tacon et al., 2009; Wiseman et al., 2014).

**Table S16**. Levels and schematic representations of attributes for DCE1

| Section | Icon | Label | Description |
| --- | --- | --- | --- |
| Feed conversion rate | 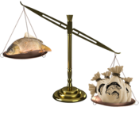 | 5 kg feed to 1 kg fish | • Fish requires the normal amount of feed to grow |
|  | 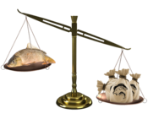 | 4.5 kg feed to 1 kg fish | • Fish requires a little less feed to grow (4.5 kg of feed to get 1 kg of fish) |
|  | 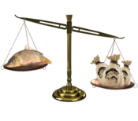 | 4 kg feed to 1 kg fish | • Fish requires less feed to grow (4 kg of feed to get 1 kg of fish) |
|  | 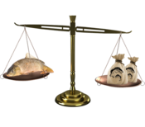 | 3.5 kg feed to 1 kg fish | • Fish requires significantly less feed to grow (3.5 kg of feed to get 1 kg of fish) |
| Survival rate | 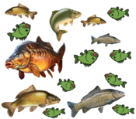 | 40% survival | • The percentage of fish loss remains normal, about 60% loss |
|  | 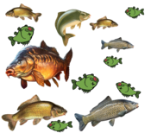 | 50% survival | • The percentage of fish loss is reduced a little, about 50% loss |
|  | 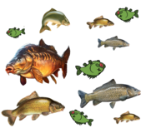 | 60% survival | • The percentage of fish loss is reduced, about 40% loss |
|  | 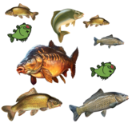 | 70% survival | • The percentage of fish loss is significantly reduced, only about 30% loss |
| Input cost | 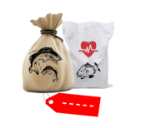 | Input cost unchanged | • The cost of fish feed will remain the same |
|  | 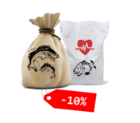 | 10% lower input cost | • The cost of fish feed will be reduced a little to about 10% less |
|  | 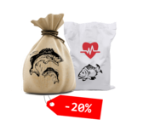 | 20% lower input cost | • The cost of fish feed will be reduced to 20% less |
|  | 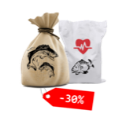 | 30% lower input cost | • The cost of fish feed will be reduced significantly to 30% less |
| Growth rate | 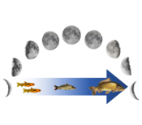 | No Change (2.5 years) | • The time needed to grow fish that is ready to be consumed will remain the same (around 2.5 years) |
|  | 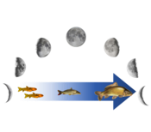 | 2 months faster | • The time needed to grow fish that is ready to be consumed will be reduce a little (around 2 years and 4 months) |
|  | 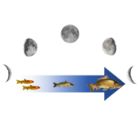 | 4 months faster | • The time needed to grow fish that is ready to be consumed will be reduce (around 2 years and 2 months) |
|  | 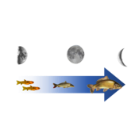 | 6 months faster | • The time needed to grow fish that is ready to be consumed will be reduce significantly (around 2 years) |

**Table S17**. Levels and schematic representations of attributes for DCE2

| Section | Icon | Label | Description |
| --- | --- | --- | --- |
| Fish bone density | 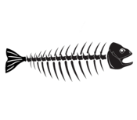 | No change (many bones) | • Grown fish will have the same amount of bones |
|  | 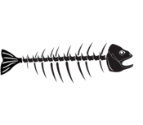 | 20% less bones | • Grown fish will have a little less amount of bones (around 20% less bones) |
|  | 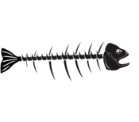 | 40% less bones | • Grown fish will have less amount of bones (around 40% less bones) |
|  | 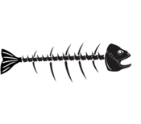 | 60% less bones | • Grown fish will have significantly less amount of bones (around 60% less bones) |
| Fish fat content | 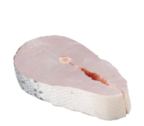 | No change | • Grown fish will have no improved taste or fat content |
|  | 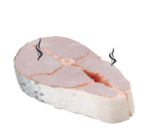 | 20% more fat | • Grown fish will have a little improved taste with 20% more fat content |
|  | 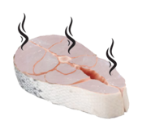 | 40% more fat | • Grown fish will have improved taste with 40% more fat content |
|  | 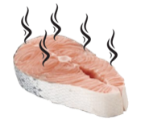 | 60% more fat | • Grown fish will have significantly improved taste with 60% more fat content |
| Fish appearance | 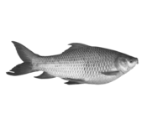 | No change (silver) | • Grown fish will have improved appearance |
|  | 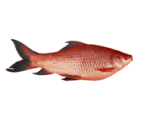 | Vivid red | • Grown fish will have an improved appearance and a vivid red color |
|  | 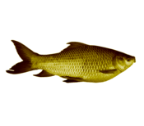 | Vivid yellow | • Grown fish will have an improved appearance and a vivid yellow color |
|  | 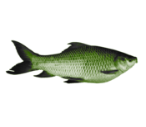 | Vivid green | • Grown fish will have an improved appearance and a vivid green color |
| Fish size | 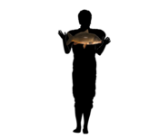 | No change (3 kg) | • The average size of fish harvested will remain the same (around 3kg) |
|  | 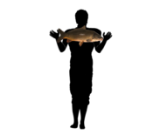 | Small improvement (4 kg) | • The average size of fish harvested will increase a little (around 4kg) |
|  | 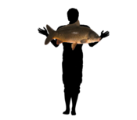 | Medium improvement (5 kg) | • The average size of fish harvested will increase (around 5kg) |
|  | 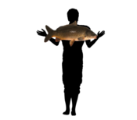 | High improvement (6 kg) | • The average size of fish harvested will increase significantly (around 6kg) |

**Table S18**. Levels and schematic representations of attributes for DCE3

| Section | Icon | Label | Description |
| --- | --- | --- | --- |
| Labor intensity | 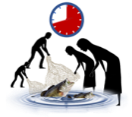 | High labor (10 days-person/decimal/year) | • Ponds will need normal labor time of 10 days-person per decimal per year |
|  | 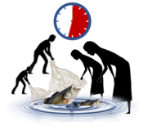 | Medium labor (8 days-person/decimal/year) | • Ponds will need little less labor time of 8 days-person per decimal per year |
|  | 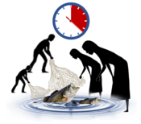 | Low labor (6 days-person/decimal/year) | • Ponds will need less labor time of 6 days-person per decimal per year |
|  | 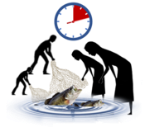 | Minimal labor (4 days-person/decimal/year) | • Ponds will need significantly less labor time of 4 days-person per decimal per year |
| Ecosystem integration | 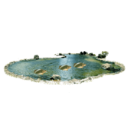 | No change (Fish pond only) | • No integration with other food system using only the ponds to grow fish |
|  | 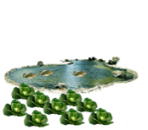 | Some ecosystem management | • Some integration with other food systems such as agricultural crops that supplement the ponds |
|  | 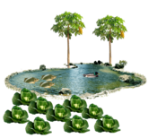 | Medium ecosystem management | • Medium integration with other food systems including agricultural and small livestock that supplement the ponds |
|  | 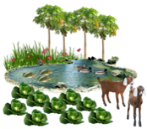 | High ecosystem management | • High integration with other food systems including agricultural and livestock that supplement the ponds |
| Government support | 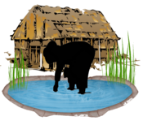 | No change (no external support) | • No new support from government institutions |
|  | 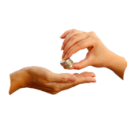 | Higher access to credit | • Increase support from the government with more access to credit |
|  | 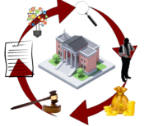 | Higher access to extension services | • Increase support from the government with more extension services |
|  | 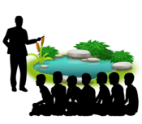 | Higher access to trainings | • Increase support from the government with more trainings |
| Management system | 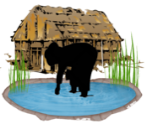 | No change (individual household) | • Individual household management with no support from associations or production groups |
|  | 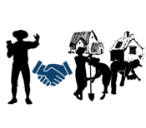 | Co-management with community | • Pond management support with community groups |
|  | 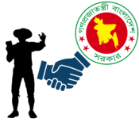 | Co-management with government | • Pond management support with government institutions |
|  | 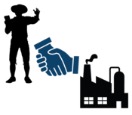 | Co-management with private sector | • Pond management support with the private sectors |

**SI References**

Agboola, J.O., Verreth, J., Yossa, R., 2019. Assessment of existing and potential feed resources for improving aquaculture production in selected Asian and African countries. https://doi.org/10.13140/RG.2.2.34156.72323

Ahme, N., 2013. On-farm feed management practices for Nile tilapia (Oreochromis niloticus) in Ghana. FAO Fisheries and Aquaculture Technical Paper 583, 191–211.

Alkire, S., Santos, M.E., 2014. Measuring Acute Poverty in the Developing World: Robustness and Scope of the Multidimensional Poverty Index. World Development 59, 251–274. https://doi.org/10.1016/j.worlddev.2014.01.026

Bangladesh - Contour Lines, 2018. https://data.humdata.org/dataset/bangladesh-contour-lines

Bangladesh - Population density, 2015. https://energydata.info/dataset/bangladesh--population-density-2015

Bangladesh - Rivers, 2018. https://data.humdata.org/dataset/bangladesh-water-courses

Bangladesh - Roads, 2018. https://data.humdata.org/dataset/bangladesh-roads

Barman, B.K., Karim, M., 2007. Analysis of feeds and fertilizers for sustainable aquaculture development in Bangladesh. FAO fisheries technical paper, Rome.

Berg, H., Mulokozi, D., Udikas, L., 2021. A GIS Assessment of the Suitability of Tilapia and Clarias Pond Farming in Tanzania. IJGI 10, 354. https://doi.org/10.3390/ijgi10050354

Bogard, J.R., Thilsted, S.H., Marks, G.C., Wahab, Md.A., Hossain, M.A.R., Jakobsen, J., Stangoulis, J., 2015. Nutrient composition of important fish species in Bangladesh and potential contribution to recommended nutrient intakes. Journal of Food Composition and Analysis 42, 120–133. https://doi.org/10.1016/j.jfca.2015.03.002

DoF, 2022. Yearbook of Fisheries Statistics of Bangladesh, 2020-21. Department of Fisheries. Bangladesh: Ministry of Fisheries and Livestock.

Donovan, C., Damaseke, M., Govereh, J., Simumba, D., Donovan, C., Damaseke, M., Govereh, J., Simumba, D., 2000. Framework and Initial Analyses of Fertilizer Profitability in Maize and Cotton in Zambia. https://doi.org/10.22004/AG.ECON.54460

Falconer, L., Telfer, T.C., Ross, L.G., 2016. Investigation of a novel approach for aquaculture site selection. Journal of Environmental Management 181, 791–804. https://doi.org/10.1016/j.jenvman.2016.07.018

Ghobadi, M., Nasri, M., Ahmadipari, M., 2021. Land suitability assessment (LSA) for aquaculture site selection via an integrated GIS-DANP multi-criteria method; a case study of lorestan province, Iran. Aquaculture 530, 735776. https://doi.org/10.1016/j.aquaculture.2020.735776

Gong, P., Liu, H., Zhang, M., Li, C., Wang, J., Huang, H., Clinton, N., Ji, L., Li, Wenyu, Bai, Y., Chen, B., Xu, B., Zhu, Z., Yuan, C., Ping Suen, H., Guo, J., Xu, N., Li, Weijia, Zhao, Y., Yang, J., Yu, C., Wang, X., Fu, H., Yu, L., Dronova, I., Hui, F., Cheng, X., Shi, X., Xiao, F., Liu, Q., Song, L., 2019. Stable classification with limited sample: transferring a 30-m resolution sample set collected in 2015 to mapping 10-m resolution global land cover in 2017. Science Bulletin 64, 370–373. https://doi.org/10.1016/j.scib.2019.03.002

Green, B.W., 2015. Fertilizers in aquaculture, in: Feed and Feeding Practices in Aquaculture. Elsevier, pp. 27–52. https://doi.org/10.1016/B978-0-08-100506-4.00002-7

Hossain, M.S., Chowdhury, S.R., Das, N.G., Sharifuzzaman, S.M., Sultana, A., 2009. Integration of GIS and multicriteria decision analysis for urban aquaculture development in Bangladesh. Landscape and Urban Planning 90, 119–133. https://doi.org/10.1016/j.landurbplan.2008.10.020

Jayanthi, M., Thirumurthy, S., Samynathan, M., Manimaran, K., Duraisamy, M., Muralidhar, M., 2020. Assessment of land and water ecosystems capability to support aquaculture expansion in climate-vulnerable regions using analytical hierarchy process based geospatial analysis. Journal of Environmental Management 270, 110952. https://doi.org/10.1016/j.jenvman.2020.110952

Johnston, R., 2011. Bangladesh national drinking water quality survey of 2009. UNICEF.

M. Abdelhamid, A., F. Salem, M., El-Sh. Ramadan, M., 2019. Comparison Between Effects of Sinking and Floating Diets on Growth Performance of the Nile Tilapia (Oreochromis niloticus). Egypt. J. of Aquatic Biolo. and Fish. 23, 347–361. https://doi.org/10.21608/ejabf.2019.31849

Nayak, A.K., Kumar, P., Pant, D., Mohanty, R.K., 2018. Land suitability modelling for enhancing fishery resource development in Central Himalayas (India) using GIS and multi-criteria evaluation approach. Aquacultural Engineering 83, 120–129. https://doi.org/10.1016/j.aquaeng.2018.10.003

Ng, W.K., Teh, S.W., Chowdhury, K.M., Burea, D.P., 2013. On-farm feeding and feed management in tilapia aquaculture in Malaysia. FAO Fisheries and Aquaculture Technical Paper 583, 407–431.

Rahman, M.L., Mondal, M.N., Shahin, J., 2014. Evaluation of the Quality of Commercially Manufactured Fish Feeds used for Aquaculture in Bangladesh. International Journal of Applied Research and Studies 3, 1–9.

Saba, G.K., Steinberg, D.K., 2012. Abundance, Composition and Sinking Rates of Fish Fecal Pellets in the Santa Barbara Channel. Sci Rep 2, 716. https://doi.org/10.1038/srep00716

Settaluri, V.S., Kandala, C.V.K., Puppala, N., Sundaram, J., 2012. Peanuts and Their Nutritional Aspects—A Review. FNS 03, 1644–1650. https://doi.org/10.4236/fns.2012.312215

Shaheen, N., Rahim, A.T.M., Mohiduzzaman, M., Banu, C.P., Latiful Bari, M., Tukun, A.B., Mannan, M.A., Bhattacharjee, L., Stadlmayr, B., 2013. Food Composition Table for Bangladesh. Institute of Nutrition and Food Science, University of Dhaka, Dhaka, Bangladesh.

Tacon, A.G.J., Metian, M., Hasan, M.R., 2009. Feed ingredients and fertilizers for farmed aquatic animals: sources and composition, FAO fisheries and aquaculture technical paper. Food and Agriculture Organization of the United Nations, Rome, Italy.

WFP, 2008. Food consumption analysis: calculation and use of the food consumption score in food security analysis (Technical Guidance Sheet). United Nations World Food Programme, Rome, Italy.

WIEDER, W., 2014. Regridded Harmonized World Soil Database v1.2 59.234908 MB. https://doi.org/10.3334/ORNLDAAC/1247

Wiseman, A.J.L., Finch, H.J.S., Samuel, A.M., Lane, G.P.F., Lockhart, J.A.R., 2014. Lockhart & Wiseman’s crop husbandry including grassland, Ninth edition. ed, Woodhead Publishing series in food science, technology and nutrition. Elsevier/WP, Woodhead Publishing is an imprint of Elsevier, Amsterdam.
